# CD103^+^ regulatory T cells underlie resistance to radio-immunotherapy and impair CD8^+^ T cell activation in glioblastoma

**DOI:** 10.1038/s43018-023-00547-6

**Published:** 2023-04-20

**Authors:** Luuk van Hooren, Shanna M. Handgraaf, Daan J. Kloosterman, Elham Karimi, Lotte W.H.G. van Mil, Awa A. Gassama, Beatriz Gomez Solsona, Marnix H. P. de Groot, Dieta Brandsma, Daniela F. Quail, Logan A. Walsh, Gerben R. Borst, Leila Akkari

**Affiliations:** 1 Division of Tumor Biology and Immunology, Oncode Institute, The Netherlands Cancer Institute, Amsterdam, the Netherlands; 2 Rosalind and Morris Goodman Cancer Research Centre, McGill University, Montreal, Quebec Canada; 3 Department of Human Genetics, McGill University, Montreal, Quebec Canada; 4 Department of Neuro-Oncology, Netherlands Cancer Institute-Antoni van Leeuwenhoek, Amsterdam, the Netherlands; 5 Department of Physiology, Faculty of Medicine, McGill University, Montreal, Quebec Canada; 6 Division of Cancer Sciences, School of Medical Sciences, Faculty of Biology, Medicine and Health and Manchester Cancer Research Centre, University of Manchester, Manchester, UK; 7 Department of Radiotherapy Related Research, The Christie NHS Foundation Trust, Manchester, UK

**Keywords:** Cancer therapy, CNS cancer, Cancer immunotherapy, Radiotherapy, Cancer

## Abstract

Glioblastomas are aggressive primary brain tumors with an inherent resistance to T cell-centric immunotherapy due to their low mutational burden and immunosuppressive tumor microenvironment. Here we report that fractionated radiotherapy of preclinical glioblastoma models induce a tenfold increase in T cell content. Orthogonally, spatial imaging mass cytometry shows T cell enrichment in human recurrent tumors compared with matched primary glioblastoma. In glioblastoma-bearing mice, α-PD-1 treatment applied at the peak of T cell infiltration post-radiotherapy results in a modest survival benefit compared with concurrent α-PD-1 administration. Following α-PD-1 therapy, CD103^+^ regulatory T cells (Tregs) with upregulated lipid metabolism accumulate in the tumor microenvironment, and restrain immune checkpoint blockade response by repressing CD8^+^ T cell activation. Treg targeting elicits tertiary lymphoid structure formation, enhances CD4^+^ and CD8^+^ T cell frequency and function and unleashes radio-immunotherapeutic efficacy. These results support the rational design of therapeutic regimens limiting the induction of immunosuppressive feedback pathways in the context of T cell immunotherapy in glioblastoma.

## Abstract (web summary)

Akkari and colleagues find that CD103^+^ regulatory T cells are highly abundant in the glioblastoma environment after checkpoint blockade restraining therapy response, and show therapeutic benefit for combined Treg targeting with radio-immunotherapy.

## Main

Immune checkpoint blockade (ICB) using PD-(L)1-targeting antibodies has revolutionized the treatment of various solid tumors, yet remains poorly efficient in glioblastoma^1,2^. Despite anecdotal reports of therapeutic efficacy and durable responses in a limited glioblastoma patients subset, phase III clinical trials of concurrent ICB with fractionated radiotherapy (RT) and chemotherapy did not lead to a survival benefit^3^ (CheckMate 498 and CheckMate 548). Interestingly, in resectable recurrent glioblastoma, neoadjuvant α-PD-1 administration extended survival in a phase II clinical trial^4^. Although schedule adjustment improved outcome in other solid tumor treatments^5^, optimal ICB sequence and timing remains to be examined in glioblastoma.

In contrast to the lack of therapeutic benefit in primary brain tumors, ICB responses are frequently observed in metastatic brain lesions from primary melanoma, lung or renal cell tumors^6–8^. ICB efficacy in these tumors is considered to be potentiated by their high tumor mutational burden (TMB) and corresponding high availability of neoantigens^9^. Although neoantigen presence and spatially restricted T cell clone expansion has recently been reported in patients with glioblastoma^10^, a higher TMB did not correlate with improved ICB response in primary brain tumors^11,12^. Paradoxically, low TMB is associated with increased inflammation, better ICB response and prolonged survival in patients with either primary or recurrent glioblastoma^13^. Scarce infiltration of effector lymphoid cells^14,15^ and a myeloid-dominated immunosuppressive glioblastoma tumor microenvironment (TME) contributes to the limited ICB and standard-of-care treatment efficacy^16–18^. Therefore, whether antigen availability represents a key constraint to achieving efficient ICB response in the suppressive glioblastoma TME remains to be addressed.

Although long considered immune deserts, the presence of meningeal tertiary lymphoid structures (TLS) in glioblastoma mouse models and patients has recently been reported^19^. These structures provide a site for local antigen presentation and promote T cell recruitment in the TME. While TLS presence predicts the response to ICB in a variety of solid tumors^20,21^, whether they could heighten T cell responses in primary brain cancers has not yet been examined. Importantly, the potential of TLS to unleash effector T cell (Teff) activation can be impeded by high regulatory T cell (Treg) infiltration, as Tregs regulate tumor-associated antigen (TAA) presentation and immune responses within these structures^22^. In glioblastoma, an increased Treg abundance correlates with decreased T cell cytotoxicity^23^ and inhibiting CD4^+^ T cell differentiation into Tregs in immunogenic glioblastoma models potentiates anti-tumor immune response^24^. However, treatment-induced dynamic changes in Treg content and functions and its impact on ICB therapy remain unknown in glioblastoma.

Maintaining the intrinsic potential for central nervous system T cell immune response using immune-sensitization strategies is essential to overcome the immunosuppressive glioblastoma microenvironment. RT is a pillar of glioblastoma standard-of-care and leads to immunogenic cell death and enhanced antigen availability^1^ and can function as an immune sensitizer^25^. In this Article, we explored the TME dynamics in response to radio-immunotherapy (RT + IT) in preclinical mouse models closely mimicking human glioblastoma^16,26–28^. We demonstrate that the immunosuppressive glioblastoma TME prevents ICB therapeutic response regardless of immunogenic TAA presence. We revealed that ICB dosing schedule and the immunosuppressive glioblastoma TME both impact therapeutic outcome. Specifically, CD103^+^ Tregs with upregulated lipid metabolism following α-PD-1 concurrent to RT (RT + Conc.IT) therapy restrain cytotoxic CD8^+^ T cell activity. Depleting the scarce, but potent immunosuppressive Treg population enables TLS formation, induces a cytotoxic CD8^+^ T cell response and enhances RT + IT efficacy.

## Results

### Heterogeneity of the T cell-scarce glioblastoma microenvironment

**Fig. 1:**
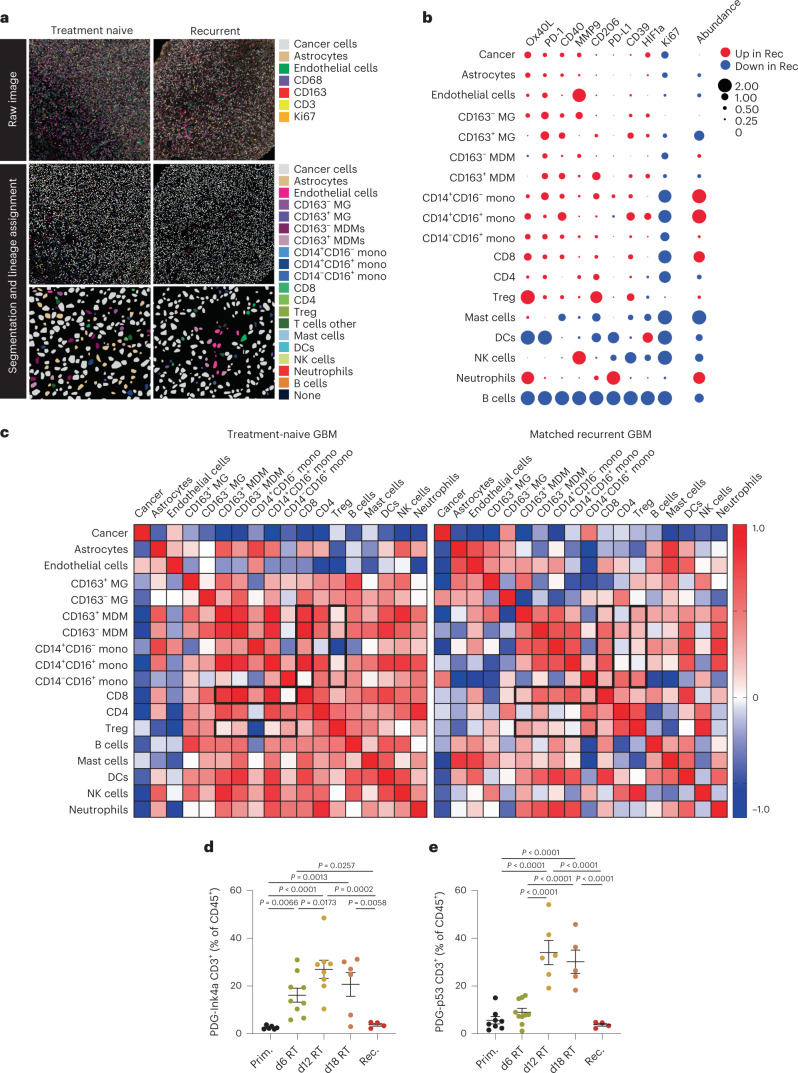
The glioblastoma microenvironment is highly heterogeneous and T cell scarce. **a**, Representative IMC images from treatment-naive human glioblastoma and their matched recurrent tumors post standard of care therapy. Unprocessed images (top) with corresponding processed images with lineage assignment (bottom) are shown, representative of *n* = 4 independent repeats. **b**, Bubble plot representing the difference in cell abundance in treatment-naive glioblastoma versus their matched recurrent tumors and the log_2_ fold change in average signal intensity of the indicated activation markers for each corresponding cell type (*n* = 4 patients). **c**, Heat map showing the Spearman correlation between indicated cell types in treatment-naive glioblastoma and their matched recurrent tumors (*n* = 4 patients). **d**,**e**, Flow cytometry quantification of CD3^+^ T cells (gated from CD45^+^CD11b^−^ cells) in the tumor microenvironment of PDG-Ink4a/Arf^-/-^ (PDG-Ink4a) (**d**) and PDG-p53^KD^ (PDG-p53) (**e**) glioblastoma isolated from primary, treatment-naive tumors (Prim) or from tumors treated with 5x2Gy RT and isolated 6 days, 12 days or 18 days post initial radiation dose (6d, 12d and 18d, respectively), or at tumor regrowth 3–4 weeks post-RT (herein termed recurrence (Rec)) (in **d**, Prim *n* = 6, d6 RT *n* = 9, d12 RT *n* = 8, d18 RT = 6, Rec *n* = 4 mice; in **e**, Prim *n* = 8, d6 RT *n* = 10, d12 RT *n* = 6, d18 RT = 5, Rec *n* = 5 mice). Statistics: one-way ANOVA with Benjamini, Krieger and Yekutieli correction for multiple testing (**d** and **e**). Data are represented as mean ± s.e.m. (**d** and **e**). Source data

**Extended Data Fig. 1:**
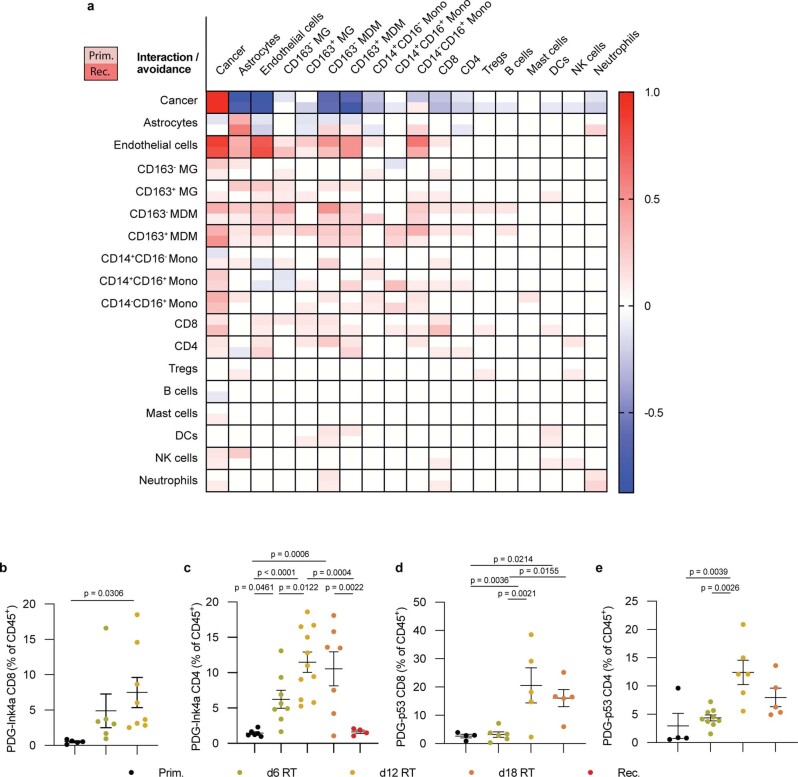
Imaging Mass Cytometry analyses of immune cell interactions and avoidance in the human glioblastoma tumor microenvironment. **a**, Heatmap showing the interaction/avoidance scores of cell types quantified in Fig. 1b in treatment-naive human glioblastoma (Primary (Prim), upper half square) and their matched recurrent tumors post standard of care therapy (Recurrent (Rec) lower half square). Each column displays the cell type interaction/avoidance score with the corresponding cell types in the rows below (n = 4 patients). **b**^**−**^**e**, Flow cytometry quantification of T cells (gated as CD45^+^CD11b^−^CD3^+^) in the TME of PDG-Ink4a (**b,c**) or PDG-p53 (**d,e**) glioblastoma isolated from treatment-naïve, primary tumors (Prim), or from tumors treated with 5x2Gy radiotherapy (RT) isolated 6 days, 12 days or 18 days post initial radiation dose (6d, 12d, 18d, respectively) or at tumor regrowth 3-4 weeks post-RT (herein termed recurrence (Rec)) tumors. **B**, CD8^+^ T cells (gated from CD45^+^CD11b^+^CD3^+^; Prim *n* = 5, d6 RT *n* = 6, d12 RT *n* = 8 mice). **c**, CD4^+^ T cells (gated from CD45^+^CD11b^+^CD3^+^; Prim *n* = 5, d6 RT *n* = 10, d12 RT *n* = 8, d18 RT *n* = 4, Rec n = 4 mice). **d**, CD8^+^ T cells (gated from CD45^+^CD11b^+^CD3^+^; Prim *n* = 4, d6 RT *n* = 6, d12 RT *n* = 5, d18 RT *n* = 5 mice). **e**, CD4^+^ T cells (gated from CD45^+^CD11b^+^CD3^+^; Prim *n* = 4, d6 RT *n* = 10, d12 RT *n* = 8, d18 RT *n* = 5 mice). Statistics: one-way ANOVA with Benjamini, Krieger and Yekutieli correction for multiple testing (**b-e**). Data are represented as mean ± S.E.M. (**b-e**). Source data

Low T cell infiltration and dominance of brain-resident microglia and monocyte-derived macrophages (MDMs) are partially responsible for limited T cell-centric immunotherapeutic efficacy in glioblastoma. Since RT can function as an immune sensitizer by inducing immunogenic cell death and increasing TAA availability^1^, we characterized the dynamic glioblastoma TME in response to RT and in recurrent tumors. We performed imaging mass cytometry (IMC) to reveal immune cell spatial localization in primary and matched recurrent human glioblastoma (Fig. 1a). As we previously reported^16^, MDM content was increased at recurrence while microglia abundance was decreased (Fig. 1b). Tumor-infiltrating CD8^+^ T cell and Treg content was enriched in relapsed tumors and exhibited elevated PD-1 and Ox40L expression (Fig. 1b). Correlation analyses showed that infiltration of monocyte and MDM within recurrent tumors was associated with increased CD8^+^ T cell and Treg infiltration (Fig. 1c). Moreover, spatial analyses revealed heightened monocyte–CD8^+^ T cell and Treg–CD8^+^ T cell interactions (Extended Data Fig. 1a), evoking functional immunosuppression. These data suggest that, despite an increased T cell content in recurrent glioblastoma, the immunosuppressive features of the recurrent TME, potentially influenced by peripherally derived myeloid cells, participate in ICB inefficiency^3^.

Human tissue analyses only permit examination of treatment-naive or relapsed glioblastoma samples, which limits longitudinal insights on the TME landscape in the course of treatment. To evaluate the dynamic immune cell response post RT, we employed two genetically engineered mouse models (GEMMs) of glioblastoma. In both models, tumor development is driven by platelet-derived growth factor-β (PDGF-B), combined with either loss of *p53* (PDG-p53) or *Ink4a/Arf* (PDG-Ink4a) (refs. ^16,26–28^). Timepoint analysis of the TME post RT (five times 2 Gy daily doses; 5x2Gy) demonstrated more than tenfold transient T cell increase in the RT response phase (Fig. 1d,e and Extended Data Fig. 1b–e). These findings show that RT induces T cell infiltration and alters the balance between myeloid and lymphoid immune compartments in the glioblastoma TME.

### The glioblastoma TME restrains ICB efficacy independently of antigen availability

**Fig. 2:**
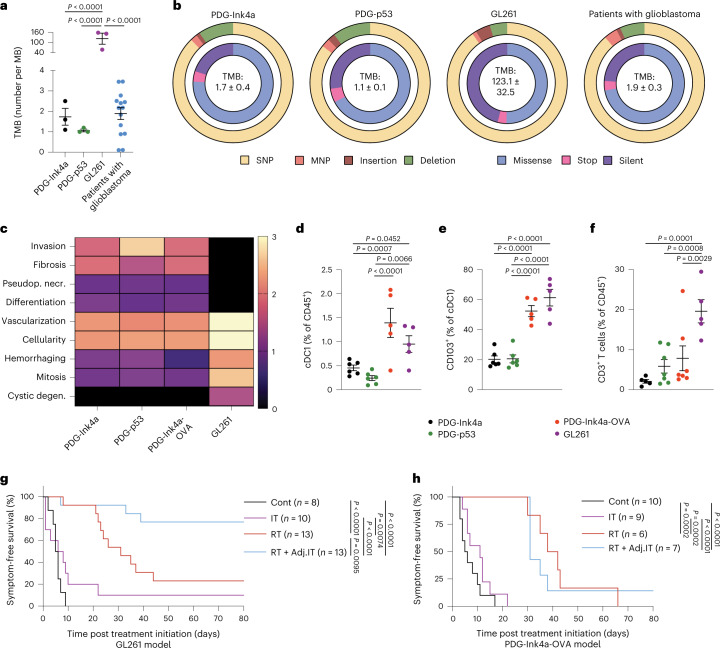
The glioblastoma tumor microenvironment rather than antigen availability restrains RT response. **a**, TMB in the PDG-Ink4a, PDG-p53 and GL261 glioblastoma mouse models and patients with glioblastoma^30^ as determined by WES analyses (Methods; MB = mutational burden; PDG-Ink4a *n* = 4 mice, PDG-p53 *n* = 3 mice, GL261 *n* = 3 mice, patients with glioblastoma *n* = 14 patients). **b**, Donut charts of the variant type (outer circle) and functional class (inner circle) distribution of mutations in each glioblastoma mouse model and from glioblastoma patient datasets^30^ (MNP = multiple nucleotide polymorphism; PDG-Ink4a control *n* = 3 mice, tumor *n* = 3 mice; PDG-p53 control *n* = 3 mice, tumor *n* = 3 mice; GL261 control *n* = 3 mice, tumor *n* = 3 mice; patients with glioblastoma *n* = 14 patients). **c**, Grading of key histopathological features observed in the PDG-Ink4a, PDG-p53, PDG-Ink4a-OVA and GL261 glioblastoma mouse models (pseudop. necr., pseudopallisading necrosis; cystic degen., cystic degeneration; PDG-Ink4a *n* = 7 mice; PDG-p53 *n* = 9 mice; PDG-Ink4a-OVA *n* = 6 mice; GL261 *n* = 9 mice). **d**–**f**, Flow cytometry quantification of CD24^+^CD11b^−^ dendritic cells (cDC1s, gated from CD45^+^Ly6C^−^CD64^−^MHCII^+^CD11c^+^ cells) (**d**), CD103^+^ cDC1s (**e**) and CD3^+^ T cells (gated from CD45^+^CD11b^−^cells) (**f**) in end-stage, treatment-naive PDG-Ink4a, PDG-p53, PDG-Ink4a-OVA and GL261 tumors (in **d** and **e**, PDG-Ink4a *n* = 6 mice, PDG-p53 *n* = 6 mice, PDG-Ink4a-OVA *n* = 5 mice, GL261 *n* = 5 mice; in **f**, PDG-Ink4a *n* = 5 mice, PDG-p53 *n* = 7 mice, PDG-Ink4a-OVA *n* = 7 mice, GL261 *n* = 5 mice). **g**,**h**, Kaplan–Meier survival curves of GL261 (**g**) and PDG-Ink4a-OVA (**h**) tumor-bearing mice treated with rIgG2a isotype control (Cont), anti-PD-1 (IT), 5x2Gy RT or adjuvant combination treatment (RT + Adj.IT). Statistics: one-way ANOVA with Benjamini, Krieger and Yekutieli correction for multiple testing (**a** and **d**–**f**), log-rank test (**g** and **h**). Data are represented as mean ± s.e.m. (**a** and **d**–**f**). Median survival and significance depicted in Supplementary Table 1 (**g** and **h**). Source data

**Extended Data Fig. 2:**
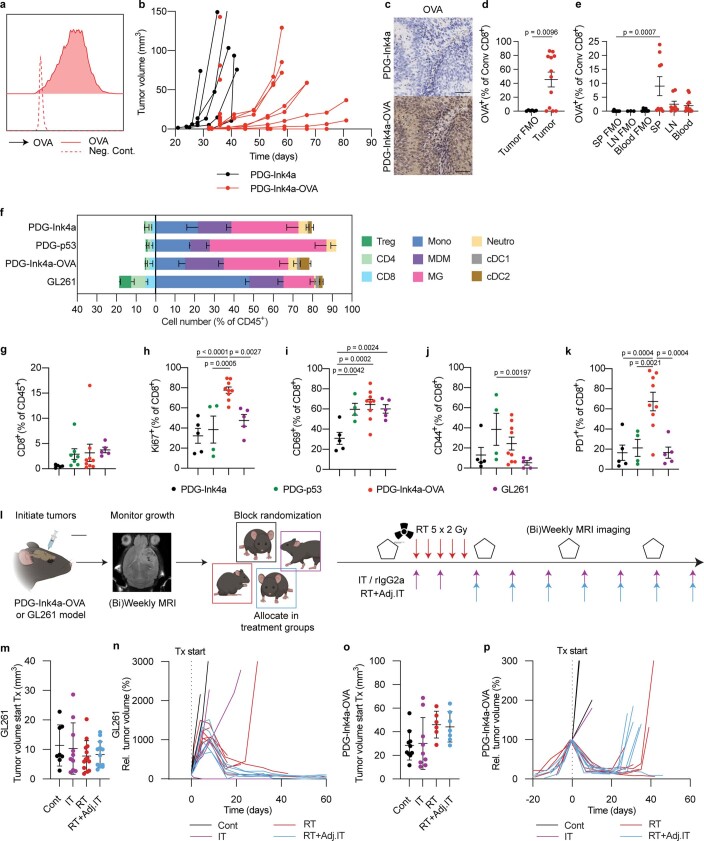
PDG-Ink4a-OVA model setup and tumor response to RT + IT in immunogenic glioblastoma. **a**, Representative flow cytometry histograms displaying ovalbumin (OVA) fluorescence intensity in DF1-OVA cells used to generate PDG-Ink4a-OVA glioblastoma. **b**, Longitudinal individual tumor volumes measured by weekly MRI in PDG-Ink4a (black) and PDG-Ink4a-OVA (red) tumor-bearing mice. Each dot is a tumor volume quantification. Lines indicate matched tumor progression per animal (PDG-Ink4a n = 6, PDG-Ink4a-OVA n = 17 mice). **c**, Representative image of immunohistochemical OVA staining in endpoint, treatment-naïve PDG-Ink4a (upper panel) and PDG-Ink4a-OVA glioblastoma (lower panel; scale bar: 50μm; representative of n = 6 PDG-Ink4a and n = 12 PDG-Ink4a-OVA independent repeats). **d**,**e** Flow cytometry quantification of OVA^+^ T cells (gated from CD45^+^CD11b^-^CD3^+^CD8^+^) in tumor (**d**) and superficial cervical lymph nodes (LN), spleen (SP) and blood (**e**) in end^−^stage, treatment-naive PDG-Ink4a-OVA tumor-bearing mice (FMO = fluorescence minus one; **d**: Tumor FMO n = 6, Tumor = 12 mice. **e**: SP FMO n = 8, LN FMO n = 3, Blood FMO n = 6, SP n = 9, LN n = 8, Blood n = 12 mice). **f**, Relative immune composition in the glioblastoma TME of primary, treatment-naive tumors. Treg = regulatory T cells, CD8 = CD8^+^ T cells, CD4 = CD4^+^ T cells, Mono = Ly6C^+^ monocytes, MDM = CD49d^+^ monocyte-derived macrophages, MG = CD49d^-^ microglia, Neutro = Ly6G^+^ neutrophils, cDC1 = CD24^+^CD11b^−^dendritic cells, cDC2 = CD24^−^CD11b^+^ dendritic cells (PDG-Ink4a: CD8 n = 2, CD4 n = 7, Treg n = 7, Mono n = 6, MDM n = 6, MG n = 6, Neutro n = 6, cDC1 n = 5, cDC2 n = 5; PDG-p53: CD8 n = 4, CD4 n = 8, Treg n = 8, Mono n = 2, MDM n = 2, MG n = 2, Neutro n = 2, cDC1, cDC2 = N/A; PDG-Ink4a-OVA: CD8 n = 9, CD4 n = 9, Treg n = 9, Mono n = 9, MDM n = 9, MG n = 9, Neutro n = 9, cDC1 n = 9, cDC2 n = 9; GL261: CD8 n = 5, CD4 n = 5, Treg n = 6, Mono n = 5, MDM n = 5, MG n = 5, Neutro n = 5, cDC1 n = 5, cDC2 n = 5). **g-k**, Flow cytometry quantification of CD8^+^ T cells in end-stage, treatment-naive PDG-Ink4a, PDG-p53, PDG-Ink4a-OVA and GL261 tumors. **g**, total CD8^+^ T cells (gated from CD45^+^CD11b^−^CD3^+^ cells). **h**, Ki67^+^ CD8^+^ T cells from (**g**). **i**, CD69^+^ CD8^+^ T cells from (**g**). **j**, CD44^+^ CD8^+^ T cells from (**g**). **k**, PD-1^+^ CD8^+^ T cells from (**g**). For **g**: PDG-Ink4a n = 5, PDG-p53 n = 7, PDG-Ink4a-OVA n = 9 mice, GL261 n = 5 mice. For **h-k**: PDG-Ink4a n = 5, PDG-p53 n = 4, PDG-Ink4a-OVA n = 9 mice, GL261 n = 5 mice. **l**, Schematic overview of the experimental design. GL261 and PDG-Ink4a-OVA tumors were initiated as described in Methods. Tumor size was quantified by MRI. Based on tumor volume, mice were distributed into treatment groups by block randomization (rIgG2a isotype control (Cont), anti-PD-1 (IT), 5x2Gy radiotherapy (RT), or adjuvant combination treatment (RT + Adj.IT)), followed^−^up weekly by MRI and sacrificed at 80d or at humane endpoint. Schematic created using BioRender.com. **m**, Distribution of GL261 tumor volume at the time of inclusion into treatment (Cont n = 8, IT n = 10, RT n = 13, RT + Adj.IT n = 13 mice). **n**, Longitudinal individual tumor volumes measured by weekly MRI in Cont, RT, IT, and RT + Adj.IT treated GL261 tumor-bearing mice (Cont n = 4, IT n = 7, RT n = 7, RT + Adj.IT n = 6 mice). **o**, Distribution of PDG-Ink4a-OVA tumor volume at the time of inclusion into treatment (Cont n = 10, IT n = 9, RT n = 6, RT + Adj.IT n = 7 mice). **p**, Longitudinal individual tumor volumes measured by weekly MRI in Cont, RT, IT and RT + Adj.IT treated PDG-Ink4a-OVA tumor-bearing mice (Cont n = 5, IT n = 4, RT n = 6, RT + Adj.IT n = 7 mice). For (**n,p**), each line indicates matched tumor progression per mouse. The vertical dashed line indicates start of treatment (Tx start). Statistics: Two-sided unpaired t-test (**d**), one-way ANOVA with Benjamini, Krieger and Yekutieli correction for multiple testing (**e,g-k**). Data are represented as mean ± S.E.M. (**d,e,g-k,m,o**) or - S.E.M. (**f**). Source data

Although an increase of CD3^+^ T cells was observed post RT and at recurrence in human tumors (Fig. 1b–d), the glioblastoma TMB has been proposed to restrict T cell responses, due to the lack of antigens T cells could respond to^9,29^. To address the relevance of TAA availability versus TMB in glioblastoma RT + IT response, we performed whole-exome sequencing (WES) in PDG-driven glioblastoma GEMMs and compared their TMB with the transplantable GL261 model and to human glioblastoma WES datasets^30^. Noteworthily, contradicting studies on the GL261 model accuracy as a representative human glioblastoma have been reported^31,32^. Indeed, GL261 tumors are immune-active^33–35^, and their therapeutic response to ICB is highly variable in literature^36–38^. The PDG models have been shown to mimic glioblastoma pathology and the clinically observed therapeutic response in patients^16,26–28^. In line with the updated World Health Organization definition for glioblastoma^39^, the PDG-driven and GL261 models are IDHwt^26–28^. As in patients with glioblastoma, both the PDG-Ink4a (1.7 ± 0.4) and PDG-p53 (1.1 ± 0.1) models displayed a low TMB (Fig. 2a) while GL261 tumors had an exceptionally high TMB (123.1 ± 32.5) and relatively high number of silent mutations and single-nucleotide polymorphisms (SNPs) (Fig. 2b). Given these findings and its equivocal response to ICB, we used the GL261 model as an example of ‘immunogenic’ glioblastoma. In addition, we adapted the PDG-Ink4a model to express the model antigen chicken ovalbumin (OVA) in cancer cells, thereby generating an immunogenic PDG-driven GEMM (PDG-Ink4a-OVA; Extended Data Fig. 2a). PDG-Ink4a-OVA outgrowth occurred with 80% penetrance, albeit with a longer latency and less aggressive pattern than the established PDG-Ink4a model (Extended Data Fig. 2b). OVA was homogeneously expressed in fully developed PDG-Ink4a-OVA tumors (Extended Data Fig. 2c), and approximately half of the infiltrating CD8^+^ T cells were OVA specific (Extended Data Fig. 2d). OVA specificity was also observed in spleen, tumor-draining superficial cervical lymph nodes (LNs) and blood-derived CD8^+^ T cells of PDG-Ink4a-OVA glioblastoma-bearing mice (Extended Data Fig. 2e).

To establish the relevance of the different glioblastoma models in relation to the human disease, we performed histopathological analyses of PDG-driven and GL261 tumors (Fig. 2c). All three PDG GEMMs displayed characteristic glioblastoma features, including pseudopallisading necrosis and a high degree of vascularization and invasive growth (Fig. 2c). In contrast, GL261 tumors exhibited high cancer cell differentiation features and lacked several of the typical human characteristics, including invasive growth and pseudopallisading necrosis (Fig. 2c). Altogether these results highlight the relevance of PDG-driven glioblastoma GEMMs, which recapitulate key genetic and histopathologic features of human glioblastoma, while GL261 tumors do not.

Flow cytometry analysis revealed that the before-mentioned pathological differences were associated with distinctive immune landscapes, with GL261 tumors presenting low tissue-resident microglia content and high monocytic infiltration (Extended Data Fig. 2f). Compared with the PDG-Ink4a and PDG-p53 models, the immunogenic GL261 and PDG-Ink4a-OVA models displayed increased CD24^+^CD11b^−^ type 1 conventional dendritic cell (cDC1) content (Fig. 2d), with heightened CD103 expression (Fig. 2e), a migratory DC marker critical for mounting a cytotoxic T cell response^40^. Examination of glioblastoma T cell infiltration showed that PDG GEMMs displayed low CD3^+^ T cell content, as seen in patients with glioblastoma^14^, which contrasted with GL261 enriched T cell numbers (Fig. 2f). We next compared the cytotoxic CD8 T cell content and activation features in the different murine models (Extended Data Fig. 2g–k). Activation and exhaustion features were heightened in Teff cells from PDG-Ink4a-OVA tumors only, with increased proliferative capacity (Extended Data Fig. 2h), activation (Extended Data Fig. 2i) and PD-1 levels (Extended Data Fig. 2k). These findings revealed that GL261 and PDG-Ink4a-OVA tumors have a high antigen availability and corresponding CD103^+^ cDC1 infiltration. However, unlike the GL261 model, PDG-Ink4a-OVA tumors show more resemblance to human glioblastoma in terms of key pathological features and T cell infiltration, representing a relevant model to study the impact of antigen availability on T cell-centric ICB response in glioblastoma.

We next evaluated the therapeutic response to RT + IT in the GL261 and PDG-Ink4a-OVA models, to address the role of the TME and high antigen availability on ICB efficacy. Glioblastoma-bearing mice were treated with RT, α-PD-1 immunotherapy (IT), or a combination of both treatments (Extended Data Fig. 2l). While RT induced glioblastoma regression and stable disease in both models, the majority of tumors ultimately regrew 3–4 weeks post RT (herein termed recurrence; Fig. 2g,h and Extended Data Fig. 2m–p). Whereas combined RT + IT yielded significant therapeutic benefit in the GL261 model, with 77% of mice presenting long-term responses (Fig. 2g, Extended Data Fig. 2m,n and Supplementary Table 1), this regimen did not induce a survival benefit in PDG-Ink4a-OVA mice (Fig. 2h, Extended Data Fig. 2o,p and Supplementary Table 1), despite the presence of proliferating and activated CD8^+^ T cells (Extended Data Fig. 2h,i). These results indicate that enforcing antigen availability in glioblastoma does not unleash therapeutic efficacy of T cell-centric IT, and suggests that the immunosuppressive TME itself may restrict ICB anti-tumor response in glioblastoma.

### RT sequence modulates immune response and survival

**Fig. 3:**
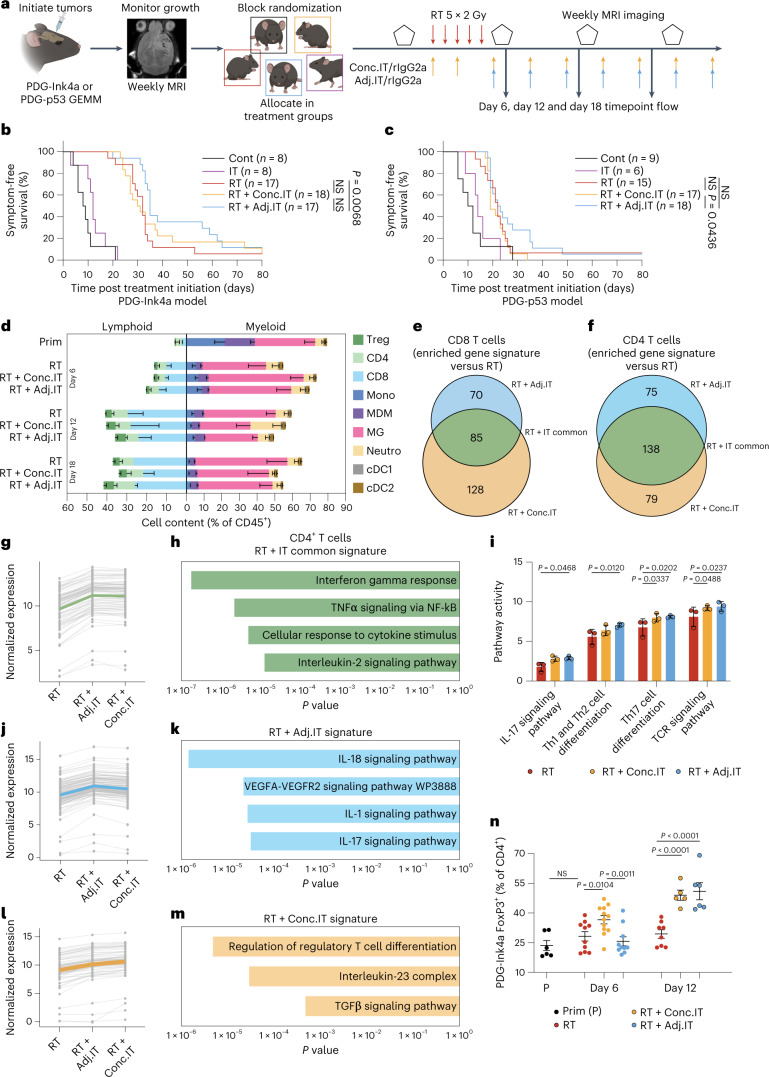
RT with adjuvant IT leads to a modest therapeutic benefit over concurrent IT in poorly immunogenic glioblastomas. **a**, Schematic overview of the experimental design. PDG-Ink4a and PDG-p53 tumors were initiated as described in Methods. At 4–7 weeks post tumor initiation, tumor size was quantified by MRI. On the basis of tumor volume, mice were distributed into treatment groups by block randomization (Cont, RT, IT, RT + Adj.IT or concurrent combination treatment (RT + Conc.IT)), followed up weekly by MRI and killed at 80 days or at humane endpoint. The schematic was created using BioRender.com. **b**,**c**, Kaplan–Meier survival curves of PDG-Ink4a-treated (**b**) and PDG-p53-treated (**c**) tumor-bearing mice. **d**, Immune composition of PDG-Ink4a tumors. Prim, primary; Treg, regulatory T cells; CD8, CD8^+^ T cells; CD4, CD4^+^ T cells; Mono, Ly6C^+^ monocytes; MDM, CD49d^+^ Ms; MG, CD49d^−^ microglia; Neutro, Ly6G^+^ neutrophils; cDC1, CD24^+^CD11b^−^ dendritic cells; cDC2, CD24^−^CD11b^+^ dendritic cells (Prim: CD8 *n* = 2, CD4 *n* = 7, Treg *n* = 7, Mono *n* = 6, MDM *n* = 6, MG *n* = 6, Neutro *n* = 6, cDC1 *n* = 5, cDC2 *n* = 5; d6 RT: CD8 *n* = 3, CD4 *n* = 8, Treg *n* = 8, Mono *n* = 7, MDM *n* = 7, MG *n* = 7, Neutro *n* = 7, cDC1 *n* = 6, cDC2 *n* = 6; d12 RT: CD8 *n* = 3, CD4 *n* = 11, Treg *n* = 11, Mono *n* = 11, MDM *n* = 11, MG *n* = 11, Neutro *n* = 11, cDC1 *n* = 10, cDC2 *n* = 10; d18 RT: CD8 *n* = 1, CD4 *n* = 7, Treg *n* = 7, Mono *n* = 6, MDM *n* = 6, MG *n* = 6, Neutro *n* = 6, cDC1 *n* = 3, cDC2 *n* = 3 mice; d6 RT + Conc.IT: CD8 *n* = 5, CD4 *n* = 10, Treg *n* = 10, Mono *n* = 7, MDM *n* = 7, MG *n* = 7, Neutro *n* = 7, cDC1 *n* = 9, cDC2 *n* = 9; d12 RT + Conc.IT: CD8 *n* = 3, CD4 *n* = 8, Treg *n* = 8, Mono *n* = 8, MDM *n* = 8, MG *n* = 8, Neutro *n* = 8, cDC1 *n* = 7, cDC2 *n* = 7; d18 RT + Conc.IT: CD8 *n* = 3, CD4 *n* = 8, Treg *n* = 8, Mono *n* = 5, MDM *n* = 5, MG *n* = 5, Neutro *n* = 5, cDC1 *n* = 3, cDC2 *n* = 3; d6 RT + Adj.IT: CD8 *n* = 3, CD4 *n* = 9, Treg *n* = 9, Mono *n* = 8, MDM *n* = 8, MG *n* = 8, Neutro *n* = 8, cDC1 *n* = 6, cDC2 *n* = 6; d12 RT + Adj.IT: CD8 *n* = 3, CD4 *n* = 9, Treg *n* = 9, Mono *n* = 9, MDM *n* = 9, MG *n* = 9, Neutro *n* = 9, cDC1 *n* = 10, cDC2 *n* = 10; d18 RT + Adj.IT: CD8 *n* = 2, CD4 *n* = 7, Treg *n* = 7, Mono *n* = 5, MDM *n* = 5, MG *n* = 5, Neutro *n* = 5, cDC1 *n* = 3, cDC2 *n* = 3). **e**,**f**, Venn diagram depicting the genes enriched in CD8^+^ (**e**) and CD4^+^ (**f**) T cells FACS-purified from d12 RT + Conc.IT and RT + Adj.IT versus RT PDG-Ink4a glioblastoma subjected to RNA-seq (Supplementary Table 2). **g**, Line charts displaying the normalized gene expression of the RT + IT common gene signatures in CD4^+^ T cells with each dot representing a gene, and lines connecting the same gene across treatment groups. Colored lines are the average of the whole gene signature (Supplementary Tables 2 and 6). **h**, Bar plots showing the adjusted *P* value of relevant significantly enriched gene sets in the RT + IT common gene signature from **g** (Supplementary Table 6). **i**, Bar plots depicting the GAGE^81^ gene set activity in CD4^+^ T cells for RT, RT + Conc.IT and RT + Adj.IT treatment groups. **j**, Line charts as described in **g** displaying the normalized gene expression of the RT + Adj.IT gene signatures in CD4^+^ T cells (Supplementary Tables 2 and 7). **k**, Bar plot showing the adjusted *P* value of relevant significantly enriched pathways in the RT + Adj.IT gene signature from **j** (Supplementary Table 7). **l**, Line charts as described in **g** displaying the normalized gene expression of the RT + Conc.IT gene signatures in CD4^+^ T cells (Supplementary Tables 2 and 8). **m**, Bar plot showing the adjusted *P* value of relevant significantly enriched pathways in the RT + Conc.IT gene signature from **l** (Supplementary Table 8). For **e**–**m**, CD4^+^ and CD8^+^ RT *n* = 3, CD4^+^ and CD8^+^ RT + Conc.IT *n* = 3, CD4^+^ and CD8^+^ RT + Adj.IT *n* = 3 mice. **n**, Flow cytometry quantification of FOXP3^+^ Tregs (gated from CD45^+^CD11b^−^CD3^+^CD4^+^ T cells) in the TME of PDG-Ink4a glioblastoma post treatment (Prim *n* = 6, d6 RT *n* = 10, d12 RT *n* = 12, d6 RT + Conc.IT *n* = 10, d12 RT + Conc.IT *n* = 8, d6 RT + Adj.IT *n* = 5, d12 RT + Adj.IT *n* = 6 mice). Statistics: Fisher’s exact test in combination with the Benjamini–Hochberg method for correction of multiple hypotheses testing (two-sided; **h**, **k** and **m**; Supplementary Tables 6–8) and one-way ANOVA with Benjamini, Krieger and Yekutieli correction for multiple testing (**i** and **n**). Data are shown as mean − s.e.m. (**d**), mean + s.e.m. (**i**) or mean ± s.e.m. (**n**). NS, not significant. Median survival and significance depicted in Supplementary Table 1 (**b** and **c**). Source data

**Extended Data Fig. 3:**
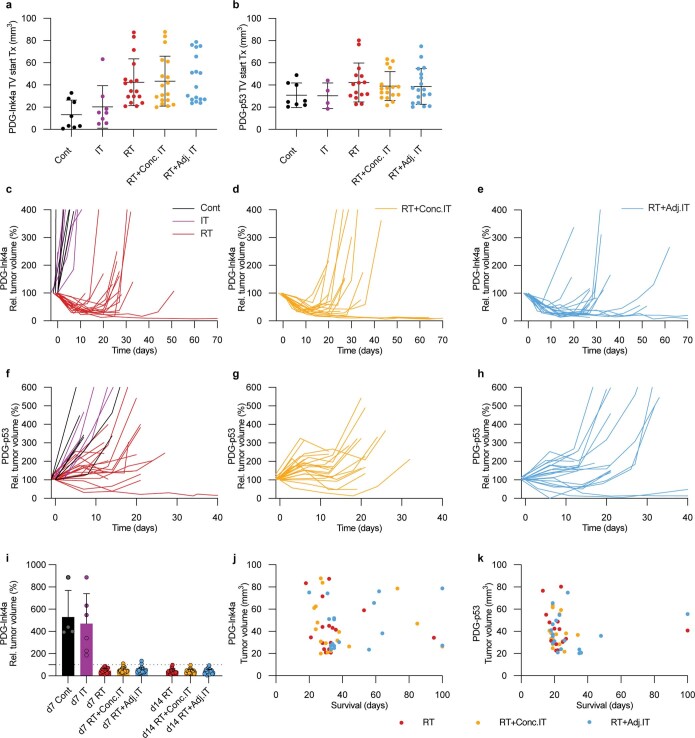
Tumor volume monitoring and progression during radio-immunotherapy response in the PDG-Ink4a and PDG-p53 poorly-immunogenic glioblastoma models. **a,b**, Distribution of PDG-Ink4a (**a**) and PDG-p53 (**b**) tumor volume measured by MRI imaging at the time of inclusion into treatment Cont, RT, IT, RT + Concurrent IT (RT + Conc.IT) and RT + Adj.IT (**a**, Cont n = 8, IT n = 8, RT n = 17, RT + Conc.IT n = 18, RT + Adj.IT n = 17 mice. **b**, Cont n = 8, IT n = 4, RT n = 15, RT + Conc.IT n = 17, RT + Adj.IT n = 18 mice). **c-h**, Longitudinal individual tumor volumes measured by weekly MRI in PDG-Ink4a (**c-e**) and PDG-p53 (**f-h**) tumor-bearing mice treated with Cont, IT, RT, RT + Conc.IT and RT + Adj.IT. Each line indicates the matched tumor progression per individual mouse (**c-e**: Cont n = 8, IT n = 8, RT n = 17, RT + Conc.IT n = 18, RT + Adj.IT n = 18 mice; **f-h**: Cont n = 8, IT n = 5, RT n = 18, RT + Conc.IT n = 22, RT + Adj.IT n = 17 mice). **i**, Tumor volume regression in PDG-Ink4a glioblastoma calculated by MRI at d7 and d14 in mice included in Cont, IT, RT, RT + Conc.IT and RT + Adj.IT treatment groups (d7 Cont n = 4, d7 IT n = 6, d7 RT n = 18, d7 RT + Conc.IT n = 22, d7 RT + Adj.IT n = 22, d14 RT n = 19, d14 RT + Conc.IT n = 23, d14 RT + Adj.IT n = 26 mice). **j,k**, Dot plot graphs depicting the correlation between PDG-Ink4a (**j**) and PDG-p53 (**k**) individual tumor volume at treatment inclusion and the animal overall survival in days (**j**: RT n = 17, RT + Conc.IT n = 17, RT + Adj.IT n = 17; **k**: RT n = 15, RT + Conc.IT n = 17, RT + Adj.IT n = 18). Data are represented as mean ± S.E.M. (**a,b**) or + S.E.M. (**i**). Source data

**Extended Data Fig. 4:**
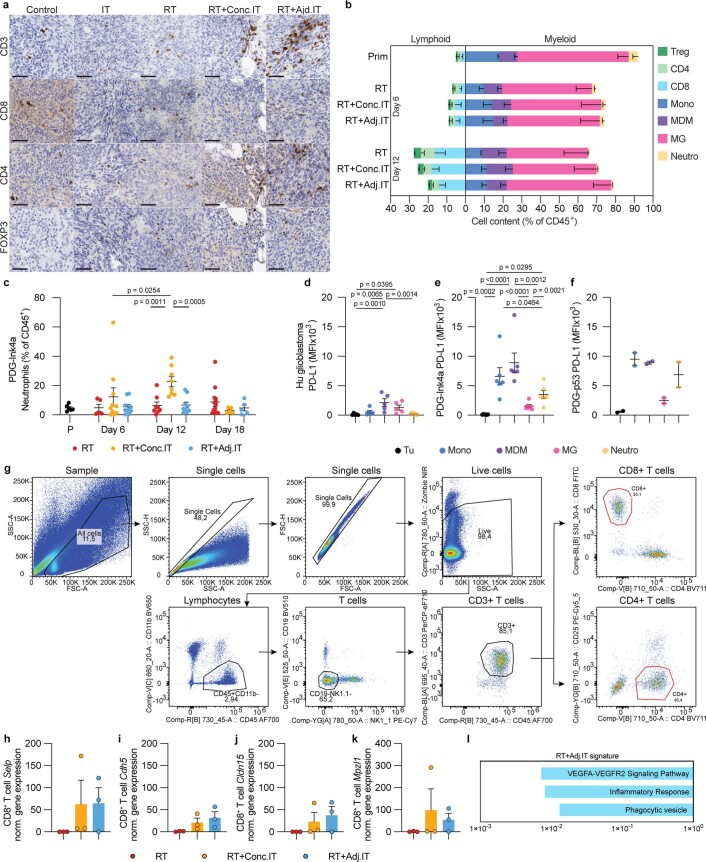
Dynamic changes in the tumor microenvironment in response to RT, RT + Conc.IT and RT + Adj.IT. **a**, Representative image of immunohistochemical staining for CD3, CD8, CD4 and FOXP3 on sequential sections of endpoint PDG-Ink4a tumors from cont, RT, IT, RT + Conc.IT and RT + Adj.IT treated mice (scale bar: 50 um; Cont is representative of n = 7, IT is representative of n = 8, RT is representative of n = 16, RT + Conc.IT is representative of n = 24 and RT + Adj.IT is representative of n = 21 independent repeats). **b**, Relative immune composition of primary (Prim) PDG-p53 glioblastoma as a percentage of CD45^+^ immune cells. Treg = regulatory T cells, CD8 = CD8^+^ T cells, CD4 = CD4^+^ T cells, Mono = Ly6C^+^ monocytes, MDM = CD49d^+^ monocyte-derived macrophages, MG = CD49d^-^ microglia, Neutro = Ly6G^+^ neutrophils (Prim: CD8 n = 4, CD4 n = 8, Treg n = 8, Mono n = 2, MDM n = 2, MG n = 2, Neutro n = 2; d6 RT: CD8 n = 6, CD4 n = 10, Treg n = 10, Mono n = 9, MDM n = 9, MG n = 9, Neutro n = 9; d12 RT: CD8 n = 4, CD4 n = 7, Treg n = 6, Mono n = 3, MDM n = 3, MG n = 3, Neutro n = 3; d6 RT + Conc.IT: CD8 n = 5, CD4 n = 9, Treg n = 9, Mono n = 9, MDM n = 9, MG n = 9, Neutro n = 9; d12 RT + Conc.IT: CD8 n = 9, CD4 n = 8, Treg n = 9, Mono n = 5, MDM n = 5, MG n = 5, Neutro n = 5; d6 RT + Adj.IT: CD8 n = 4, CD4 n = 8, Treg n = 8, Mono n = 9, MDM n = 9, MG n = 9, Neutro n = 9; d12 RT + Adj.IT: CD8 n = 5, CD4 n = 4, Treg n = 5, Mono n = 5, MDM n = 5, MG n = 5, Neutro n = 5). **c**, Flow cytometry quantification of Ly6G^+^ neutrophils (gated from CD45^+^CD11b^+^Ly6c^int^) from PDG-Ink4a treated tumors (Prim, RT, RT + Conc.IT or RT + Adj.IT) at the indicated time points post treatment initiation (Prim n = 6, d6 RT n = 5, d12 RT n = 10, d18 RT n = 10, d6 RT + Conc.IT n = 8, d12 RT + Conc.IT n = 8, d18 RT + Conc.IT n = 9, d6 RT + Adj.IT n = 12, d12 RT + Adj.IT n = 5, d18 RT + Adj.IT n = 5 mice). **d-f**, Flow cytometry quantification of PD-L1 mean fluorescence intensity (MFI) in myeloid cells in the TME of primary human (**d**), PDG-Ink4a (**e**) and PDG-p53 (**f**) glioblastoma. Tu = tumor cells (gated from CD45^−^CD11b^−^), Mono = monocytes (gated from CD45^+^CD11B^+^CD14^+^CD16^+^ (**d**) or CD45^+^CD11b^+^Ly6G^−^(**e,f**), MDM = monocyte-derived macrophages (gated from CD45^+^CD11B^+^CD14^+^CD16^−^CD49D^+^ (**d**) or CD45^+^CD11b^+^Ly6G^−^Ly6C^−^CD49d^+^ (**e,f**)), MG = microglia (gated from CD45^+^CD11B^+^CD14^+^CD16^−^CD49D^−^ (**d**) or CD45^+^CD11b^+^Ly6G^−^Ly6C^−^CD49d^−^ (**e,f**)), Neu = neutrophils (gated from CD45^+^CD11B^+^CD66B^+^ (**d**) or CD45^+^CD11b^+^Ly6G^+^Ly6C^int^ (**e,f**). **d**, n = 5 patients. **e**, n = 6 mice. **f**, n = 2 mice). **g**, Flow cytometry plots of CD4^+^ (gated CD45^+^CD11b^−^CD3^+^CD4^+^) and CD8^+^ T cell (gated CD45^+^CD11b^−^CD3^+^CD8^+^) FACS-isolation strategy of d12 RT, RT + Conc.IT and RT + Adj.IT PDG-Ink4a tumors. Sorted cells gated in red. Representative of n = 3 independent repeats. **h-k**, Normalized expression of indicated genes in CD8^+^ T cells (gated from CD45^+^CD11b^−^CD3^+^) FACS-purified from PDG-Ink4a tumors 12d post treatment initiation and subjected to RNA sequencing. **l**, Enriched pathways specific to RT + Adj.IT CD8^+^ T cells. (Supplementary Table S5). For **h-l**, RT n = 3, RT + Conc.IT n = 3 and RT + Adj.IT n = 3 mice. Statistics: one-way ANOVA with Benjamini, Krieger and Yekutieli correction for multiple testing (**c,h-k**) and Fisher’s exact test in combination with the Benjamini-Hochberg method for correction of multiple hypotheses testing (**l**). Data are represented as mean - S.E.M. (**b)**, ± S.E.M. (**c-f**) or + S.E.M. (**h-k**). Source data

We next sought to address the role of the TME in regulating IT response by treating the poorly immunogenic PDG-Ink4a and PDG-p53 models with different therapeutic regimen (Fig. 3a). On the basis of the heightened T cell infiltration observed post RT (Fig. 1d), we hypothesized that α-PD-1 treatment incorporated after RT completion (adjuvantly; RT + Adj.IT) would be superior to RT + Conc.IT, the therapeutic strategy used in unsuccessful glioblastoma clinical trials. To test this hypothesis, we allocated PDG-Ink4a and PDG-p53 glioblastoma-bearing mice (Extended Data Fig. 3a,b) into treatment groups receiving RT + Conc.IT or RT + Adj.IT (Fig. 3a and Extended Data Fig. 3c–h). In line with our previous results^16^, RT resulted in a transient tumor size regression in the PDG-Ink4a model and tumor growth stasis in the PDG-p53 model (Extended Data Fig. 3c,f). Moreover, α-PD-1 mono-IT did not improve animal survival (Fig. 3b,c, Extended Data Fig. 3c,f and Supplementary Table 1). Apart from rare long term-survivors (2/18), RT + Conc.IT treatment regimen did not result in a significant overall survival benefit compared with RT monotherapy in either model (Fig. 3b,c, Extended Data Fig. 3d,g and Supplementary Table 1). In contrast, RT + Adj.IT modestly increased overall survival in both models (Fig. 3b,c, Extended Data Fig. 3e,h and Supplementary Table 1) with neither initial tumor regression (Extended Data Fig. 3i) nor tumor size at inclusion impacting the extended survival in RT + Adj.IT long-term responders (Extended Data Fig. 3j,k). Interestingly, an increase in CD4^+^ conventional T cells (CD4^+^ T cells), but not of FOXP3^+^ regulatory T cells, was observed in RT + Adj.IT-treated endpoint mice (Extended Data Fig. 4a).

To further assess the dynamic immune response to RT + IT that may underlie the lack of Conc.IT efficacy, we performed timepoint flow cytometry analyses of tumor-infiltrating immune cells in the course of therapy response at day 6 (d6), day 12 (d12) and day 18 (d18) post-treatment initiation, in both the PDG-Ink4a and PDG-p53 models. Limited changes were observed in the myeloid compartment in response to either RT + IT regimen (Fig. 3d and Extended Data Fig. 4b) aside from an increased neutrophil content specifically in RT + Conc.IT-treated PDG-Ink4a glioblastoma (Extended Data Fig. 4c). Myeloid tumor-infiltrating cells expressed high PD-L1 levels in both murine models and glioblastoma patient samples (Extended Data Fig. 4d–f), therefore probably contributing to immune suppression^41–43^ and immunotherapy resistance^44,45^, especially since glioblastoma cells did not express PD-L1 (Extended Data Fig. 4d,e). Analyses of the lymphoid contexture in these tumors revealed that the T cell increase previously observed following RT was not significantly altered by either Conc.IT or Adj.IT (Fig. 3d and Extended Data Fig. 4b). We therefore conclude that, although an adjusted therapeutic RT + IT regimen may improve outcome, ICB is not sufficient to efficiently halt glioblastoma recurrence. A better understanding of immune cell phenotypes altered in the course of RT + IT treatment is required to devise immune-centric combinatorial approaches targeting the immunosuppressive TME and enhance ICB efficacy.

### T cell transcriptional changes in response to RT

In light of recent studies underlining that not only T cell content but also their education profiles segregate ICB responders and nonresponders^46^, we performed in-depth analyses of T cell transcriptional changes in response to RT + Conc.IT and RT + Adj.IT. We FACS-purified CD8^+^ and CD4^+^ conventional T cells from d12 PDG-Ink4a glioblastoma (Extended Data Fig. 4g), a timepoint where T cell infiltration is at its peak, and performed RNA sequencing (RNA-seq). Transcriptional analyses identified 213 upregulated genes in RT + Conc.IT and 155 in RT + Adj.IT CD8^+^ T cells, compared with RT (Fig. 3e and Supplementary Table 2). Both RT + Conc.IT and RT + Adj.IT induced comparable upregulation of cell adhesion molecule signatures (*Selp*, *Cdh5*, *Cldn15* and *Mpzl1)* in CD8^+^ T cells (Extended Data Fig. 4h–k and Supplementary Tables 2 and 3). In addition, we identified that RT + Adj.IT but not RT + Conc.IT resulted in increased angiogenic signaling signatures (*Armcx1*, *Shc2*, *Itgb5*, *Ncf2*, *P4ha2* and *Mmrn2*), inflammatory response (*Adgre1*, *Axl*, *Lif* and *Mefv*) and phagocytic vesicle signaling (*Itgb5* and *Ncf2*) in CD8^+^ T cells (Extended Data Fig. 4l and Supplementary Tables 2, 4 and 5). Nevertheless, when compared with RT, most of the transcriptional alterations identified in CD8^+^ T cells were commonly induced by both RT + IT regimen (Supplementary Tables 3–5).

**Extended Data Fig. 5:**
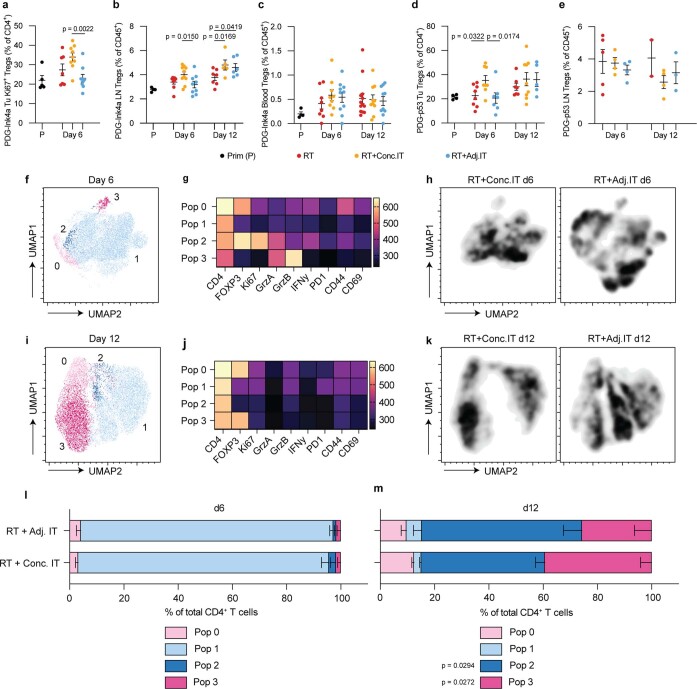
Analyses of CD4^+^ T cell and Treg subsets in radio-immunotherapy treated glioblastoma. **a-e**, Flow cytometry quantification of FOXP3^+^ Tregs from primary (P), RT, RT + Conc.IT and RT + Adj.IT treated tumor-bearing mice at indicated timepoints. **a**, Ki67^+^ Tregs (gated from CD45^+^CD11b^−^CD3^+^CD4^+^FOXP3^+^ T cells) in PDG-Ink4a tumors (Primary n = 5, RT n = 7, RT + Conc.IT n = 9, RT + Adj.IT n = 9 mice). **b**,**c** FOXP3^+^ Tregs (gated from CD45^+^CD11b^−^CD3^+^CD4^+^ T cells) in the superficial cervical lymph nodes (LN) (**b**) and blood (**c**) of PDG-Ink4a tumor bearing mice (**b**: Primary n = 4, d6 RT n = 9, d12 RT n = 10, d6 RT + Conc.IT n = 9, d12 RT + Conc.IT n = 7, d6 RT + Adj.IT n = 5, d12 RT + Adj.IT n = 6 mice. **c**: Primary n = 4, d6 RT n = 8, d12 RT n = 9, d6 RT + Conc.IT n = 10, d12 RT + Conc.IT n = 13, d6 RT + Adj.IT n = 10, d12 RT + Adj.IT n = 10 mice). **d,e** FOXP3^+^ Tregs (gated from CD45^+^CD11b^−^CD3^+^CD4^+^ T cells) in tumors (**d**) and LN (**e**) of PDG-p53 tumor-bearing mice (**d**: Primary n = 4, d6 RT n = 8, d12 RT n = 8, d6 RT + Conc.IT n = 7, d12 RT + Conc.IT n = 7, d6 RT + Adj.IT n = 9, d12 RT + Adj.IT n = 5 mice. **e**: d6 RT n = 5, d12 RT n = 4, d6 RT + Conc.IT n = 4, d12 RT + Conc.IT n = 2, d6 RT + Adj.IT n = 4, d12 RT + Adj.IT n = 3 mice). **f**, UMAP projection and unsupervised FlowSOM clustering of CD4^+^ T cell subpopulations in PDG-Ink4a glioblastoma 6d post treatment initiation identified 4 distinct subpopulations of CD4^+^ T cells (Pop 0-3). **g**, Heatmap depicting the MFI of activation markers for each subpopulation identified in (**f**). **h**, UMAP density projections plot of CD4 + T cell subpopulations from (**f**). **i**, UMAP projection and unsupervised FlowSOM clustering of CD4^+^ T cell subpopulations in PDG-Ink4a glioblastoma 12d post treatment initiation identified 4 distinct subpopulations of CD4^+^ T cells (Pop 0-3). **j**, Heatmap depicting the MFI of activation markers for each subpopulation identified in (**i**). **k**, UMAP density projections plot of CD4 + T cell subpopulations from (**i**) **l-m**, Stacked bar plot displaying the distribution of CD4^+^ T cells subpopulations in RT + Conc.IT and RT + Adj.IT treated tumors at d6 (**l**) and d12 (**m**) post treatment initiation. For **f-h,l**: d6 RT + Conc.IT n = 4, d6 RT + Adj.IT n = 4 mice. For **i-k,m**: d12 RT + Conc.IT n = 6, d12 RT + Adj.IT n = 7 mice. Statistics: one-way ANOVA with Benjamini, Krieger and Yekutieli correction for multiple testing (**a-e**) and two-stage linear step-up procedure of Benjamini, Krieger and Yekutieli (**m**). Data are represented as mean ± S.E.M. (**a-e**) or - S.E.M. (**l,m**). Source data

Gene Ontology analyses highlighted more pronounced differences in the 217 and 213 significantly upregulated genes identified in RT + Conc.IT and RT + Adj.IT CD4^+^ T cells, respectively (Fig. 3f and Supplementary Table 2). In the ‘RT + IT common’ signature, consisting of genes enriched in both RT + Conc.IT and RT + Adj.IT compared with RT CD4^+^ T cells, proinflammatory cytokine pathways (IFNγ, TNFα and IL-2 signaling) were upregulated (Fig. 3g,h and Supplementary Table 6). GAGE gene set enrichment analysis further identified upregulation of TCR signaling and Th17 differentiation pathways, while IL-17 signaling and Th1/Th2 differentiation pathways were specifically induced in RT + Adj.IT CD4^+^ T cells (Fig. 3i). Additional proinflammatory pathways were enriched in RT + Adj.IT CD4^+^ T cells only, including IL-1, IL-17, IL-18 and VEGFA-VEGFR2 signaling (Fig. 3j,k and Supplementary Table 7), the latter also observed in RT + Adj.IT CD8^+^ T cells (Extended Data Fig. 4l). The gene signature enrichment identified in RT + Conc.IT CD4^+^ T cells contrasted with their RT + Adj.IT counterpart, with distinct upregulation of pathways associated with TGFβ signaling and Treg differentiation (Fig. 3l,m and Supplementary Table 8). This latter result was supported by independent timepoint analyses showing that Treg content was heightened in both RT + Conc.IT- and RT + Adj.IT-treated tumors at d12, but increased earlier upon RT + Conc.IT treatment, with their proliferative capacity significantly elevated in the TME (Fig. 3n and Extended Data Fig. 5a). Interestingly, while Treg accumulated in the LN, no differences were observed in the systemic circulation (Extended Data Fig. 5b,c). A comparable increase of Tregs was confirmed in d6 RT + Conc.IT PDG-p53 glioblastoma (Extended Data Fig. 5d), but not in their LN (Extended Data Fig. 5e).

The difference in therapeutic response and immune composition observed between RT + Conc.IT- and RT + Adj.IT-treated mice prompted us to further analyze the CD4^+^ T cell pool at d6 and d12 post treatment (Extended Data Fig. 5f–m). In-depth spectral flow cytometry followed by uniform manifold approximation and projection (UMAP)^47^ and FlowSOM clustering analysis^48^ identified four main CD4^+^ T cell subpopulations shared among treatment groups at both timepoints (Extended Data Fig. 5f,g,i,j). At d6, limited differences between the RT + Adj.IT- or RT + Conc.IT-treated TME were observed, and naive CD4^+^ T cells (population 1; CD4^+^FOXP3^−^CD44^low^) were the most abundant subset with only limited CD4^+^ Teff and CD8^+^ cytotoxic T cells identified (population 3; CD4^+^FOXP3^−^GrzA^Int^GrzB^High^) (Extended Data Fig. 5f–h,l). The TME contexture found at d6 was however substantially altered at d12. Indeed, RT + Conc.IT treatment induced an abundance of FOXP3^+^ Treg subsets with limited activated phenotype (population 0; CD4^+^FOXP3^+^GrzB^Int^ and population 3; CD4^+^FOXP3^+^GrzB^low^; Extended Data Fig. 5i–k,m). In contrast, the RT + Adj.IT TME displayed high levels of both naive and activated conventional CD4^+^ T cells (population 2; CD4^+^FOXP3^-^CD44^low^ and population 1; CD4^+^FOXP3^-^CD44^Int^Ki67^Int^, respectively; Extended Data Fig. 5i–k,m). Altogether, these analyses indicate that RT + IT induces distinct transcriptional profiles in T cells dependent on Conc.IT or Adj.IT treatment schedules, resulting in different shaping of the glioblastoma TME. While RT + Adj.IT leads to a CD4^+^ conventional T cell abundance and an IL-18/IL-17 cytokine profile that may contribute to a proinflammatory, cytotoxic T cell response^49,50^, RT + Conc.IT results in TGFβ signaling, Treg differentiation and local proliferation. These results suggest that regulation of T cell subset content and features underlie the improved therapeutic response of RT + Adj.IT, and that the early Treg induction in RT + Conc.IT-treated glioblastoma may impair treatment efficacy.

### Treg composition and features are altered upon α-PD-1 treatment

**Fig. 4:**
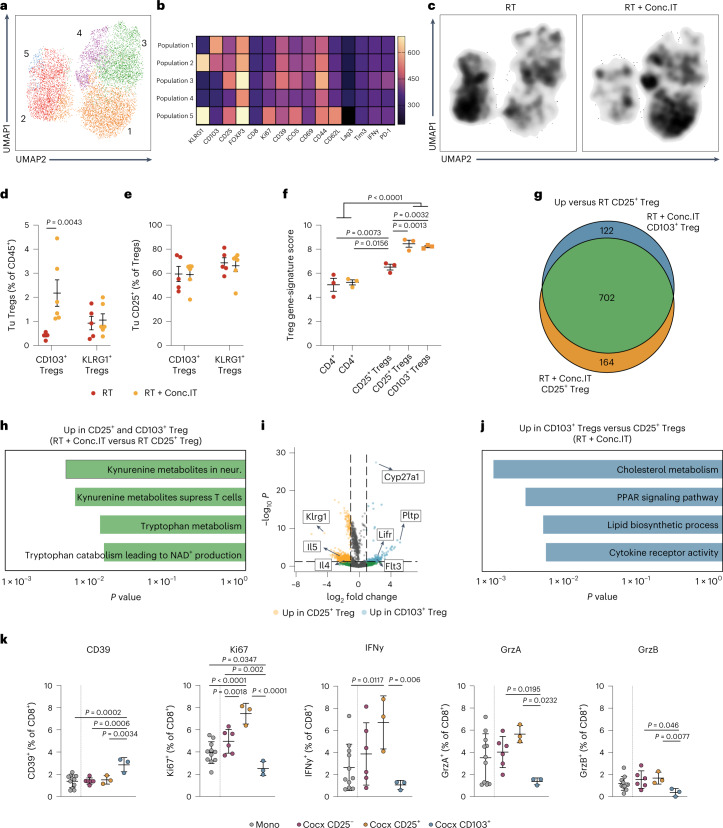
α-PD-1 checkpoint blockade alters the regulatory T cell contexture and leads to immunosuppressive CD103^+^ Tregs accumulation in the glioblastoma TME. **a**, UMAP^47^ projection and unsupervised FlowSOM^48^ clustering of the Treg population in PDG-Ink4a tumors identified five distinct subpopulations of Tregs (Pop 1–5). **b**, Heat map depicting the mean fluorescence intensity (MFI) of activation markers for the identified Treg subpopulations in **a**. **c**, UMAP density projections plot of Treg subpopulations from **a** in RT and RT + Conc.IT treatment groups. For **a**–**c**: RT *n* = 5, RT + Conc.IT *n* = 6 mice. **d**, Quantification of CD103^+^ Tregs (gated from CD45^+^CD11b^+^CD3^+^CD4^+^FOXP3^+^KLRG1^−^) and KLRG1^+^ Tregs (gated from CD45^+^CD11b^+^CD3^+^CD4^+^FOXP3^+^) in RT- or RT + Conc.IT-treated PDG-Ink4a tumors (Tu, tumor). **e**, Quantification of CD25^+^ Tregs in the CD103^+^ and KLRG1^+^ Treg populations from **d**. For **d** and **e**: RT CD103^+^ *n* = 5, RT KLRG1^+^ *n* = 5, RT + Conc.IT CD103^+^ *n* = 6, RT + Conc.IT KLRG1^+^ *n* = 6 mice. **f**–**j**, CD4^+^ T cells, CD25^+^ Tregs and CD103^+^ Tregs FACS-purified from RT- and RT + Conc.IT-treated PDG-Ink4a glioblastoma submitted to RNA-seq analyses. Enrichment of the Magnuson Treg gene signature^54^ (**f**) (RT CD4^+^ *n* = 3, RT + Conc.IT CD4^+^ *n* = 3, RT CD25^+^ *n* = 3, RT + Conc.IT CD25^+^ *n* = 3, RT + Conc.IT CD103^+^ *n* = 3 mice). Venn diagram (**g**) of differentially upregulated genes in RT + Conc.IT CD103^+^ Tregs and RT + Conc.IT CD25^+^ Tregs versus RT CD25^+^ Tregs (Supplementary Table 2). Bar graph (**h**) of upregulated pathways identified from the 702 shared genes common to RT + Conc.IT CD25^+^ Tregs and RT + Conc.IT CD103^+^ Tregs versus RT CD25^+^ Tregs (Supplementary Table 9). Volcano plot (**i**) depicting log_2_ fold change (*x* axis) versus significance (−log_10_(*P* value)) of differentially expressed genes in RT + Conc.IT CD25^+^ versus RT + Conc.IT CD103^+^ Tregs (Supplementary Table 12). Bar graph (**j**) depicting the upregulated pathways identified from the 122 genes upregulated only in RT + Conc.IT CD103^+^ Tregs (not in RT + Conc.IT CD25^+^ Tregs) versus RT CD25^+^ Tregs (Supplementary Table 11). For **g**–**j**: RT CD25^+^ *n* = 3, RT + Conc.IT CD25^+^ *n* = 3, RT + Conc.IT CD103^+^ *n* = 3 mice. **k**, Flow cytometry quantification of CD39^+^, Ki67^+^, IFNy^+^, GrzB^+^ and GrzA^+^ FACS-purified CD8^+^ T cells (from control spleens) after 24 h of monoculture (mono) or co-culture (cocx) with CD25^−^ T cells, CD25^+^ or CD103^+^ Tregs isolated from RT + Conc.IT-treated PDG-Ink4a. Cells were stimulated with anti-CD3/anti-CD28 antibodies, and cultured at a 1:1 ratio (Tregs:CD8^+^ T cells; mono: *n* = 11, CD25^−^ *n* = 6, CD25^+^ *n* = 3, CD103^+^ *n* = 3 biologically independent samples). For all graphs, analyses were done at d12 post treatment initiation on the tumor-containing brain quadrant. Statistics: one-way ANOVA with Benjamini, Krieger and Yekutieli correction for multiple testing (**d**–**f** and **k**), Fisher’s exact test (two-sided; **h** and **j**) and Wald test (**i**) in combination with the Benjamini–Hochberg method for correction of multiple hypotheses testing (two-sided; **h** and **j**). Data are represented as mean ± s.e.m. (**d**–**f**) or ± s.d. (**k**). Gating strategies (**g** and **k**) depicted in Extended Data Fig. 6a. Source data

As distinct Treg subsets with different functions can hamper Teff cell responses and immune surveillance^51^, we analyzed Treg heterogeneity and subset expansion in the context of RT + Conc.IT. We performed spectral flow cytometry, UMAP and FlowSOM clustering analyses of the lymphoid response in d12-treated glioblastoma (Fig. 3n) and identified five main subpopulations of Tregs with distinct content and profiles in the RT- and RT + Conc.IT-treated TME (Fig. 4a-b). RT + Conc.IT induced a clear shift in the abundance of these populations, with KLRG1^int^CD103^int^ Tregs (population 2; KLRG1^+^ Tregs) being the most predominant subset in RT-treated glioblastoma and a CD103^+^KLRG1^−^ Treg subpopulation (population 1; CD103^+^ Tregs) being most abundant in RT + Conc.IT-treated glioblastoma (Fig. 4c,d). Both KLRG1^+^ Tregs and CD103^+^ Tregs expressed comparable levels of CD25, a selective and targetable Treg marker^52,53^, which was not altered by RT + Conc.IT treatment (Fig. 4e).

**Extended Data Fig. 6:**
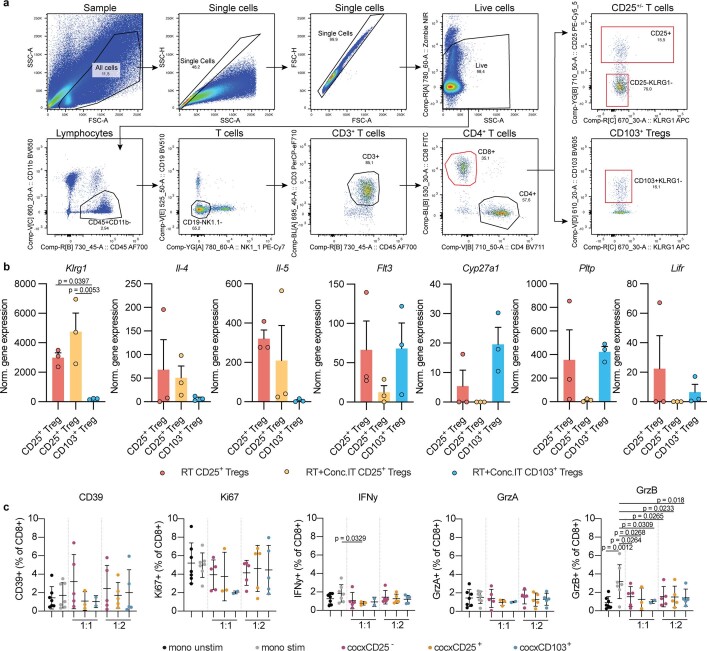
Transcriptional and functional analyses of Treg subsets in RT + Conc.IT treated glioblastoma. **a**, Representative flow cytometry plots depicting the FACS-isolation strategy for CD25^−^ T cells (gated CD45^+^CD11b^−^CD3^+^CD4^+^CD8^−^KLRG1^−^), CD25^+^ Tregs (gated CD45^+^CD11b^−^CD3^+^CD4^+^CD8^−^) and CD103^+^ Tregs (gated CD45^+^CD11b^−^CD3^+^CD4^+^CD8^−^KLRG1^−^) from tumors or spleens of PDG-Ink4a glioblastoma-bearing mice, 12d post RT and RT + Conc.IT treatment initiation, as well as CD8^+^ T cells from control spleens (gated CD45^+^CD11b^−^CD3^+^). Sorted cells gated in red. Representative of n = 5 independent repeats. **b**, Bar graphs showing the normalized expression of indicated genes in CD25^+^ Tregs (gated from CD45^+^CD11b^+^CD3^+^CD8^−^CD4^+^) and CD103^+^ Tregs (gated from CD45^+^CD11b^+^CD3^+^CD8^−^CD4^+^KLRG1^−^) FACS-purified from PDG-Ink4a glioblastoma 12d post treatment initiation (RT CD25^+^ n = 3, RT + Conc.IT CD25^+^ n = 3, RT + Conc.IT CD103^+^ n = 3 mice). **c**, Flowcytometry quantification of CD39^+^, Ki67^+^, IFNy^+^, GrzB^+^ and GrzA^+^ FACS-purified CD8^+^ T cells (CD45^+^CD11b^−^CD3^+^) from control spleen after 24 h of monoculture (mono) or co-culture (cocx) with CD25^−^ T cells (CD45^+^CD11b^−^CD3^+^CD4^+^CD8^−^KLRG1^−^), CD25^+^ Tregs (CD45^+^CD11b^−^CD3^+^CD4^+^CD8^−^) or CD103^+^ Tregs (CD45^+^CD11b^−^CD3^+^CD4^+^CD8^−^KLRG1^−^) isolated from spleens of tumor-bearing PDG-Ink4a mice 12d post RT + Conc.IT initiation. Cells were stimulated with anti-CD3/anti-CD28 antibodies, except for the monoculture control (mono unstim), and cultured in 1:1 and 1:2 ratios (Treg:CD8^+^ T cell; unstim n = 7, mono: n = 7, 1:1 CD25^−^ n = 5, 1:1 CD25^+^ n = 3, 1:1 CD103^+^ n = 2, 1:2 CD25^−^ n = 5, 1:2 CD25^+^ n = 5, 1:2 CD103^+^ n = 5 biologically independent samples). Statistics: one-way ANOVA with Benjamini, Krieger and Yekutieli correction for multiple testing (**b,c**). Data in this figure are represented as mean + S.E.M (**b**) and ± S.E.M (**c**). Gating strategies (**c**) depicted in Extended Data Fig. 6a. Source data

To characterize the CD25^+^ Treg population as a whole and the RT + Conc.IT-induced CD103^+^ Treg subset, we performed FACS purification (Extended Data Fig. 6a) and RNA-seq analyses of these partially overlapping Treg populations in independent RT + Conc.IT-treated mouse cohorts, comparing them with RT-treated CD25^+^ isolated Tregs. Transcriptional gene expression obtained from the sorted Treg populations were compared to a published pan-cancer tumor-infiltrating Treg gene signature (the Magnuson signature), which was previously validated to represent intratumoral Tregs with T cell suppressive capacity^54^. We first confirmed that CD25^+^ Tregs presented higher Magnuson signature activity than CD25^−^ conventional CD4^+^ T cells (Fig. 4f). Both CD25^+^ and CD103^+^ Treg populations isolated from RT + Conc.IT-treated tumors displayed increased Magnuson signature activity compared with RT-CD25^+^ Tregs, indicating their enhanced T cell suppressive transcriptional education. Delving into the transcriptional differences between Treg subsets, we identified 866 genes upregulated in RT + Conc.IT CD25^+^ and 824 in RT + Conc.IT CD103^+^ Tregs, when compared with RT-CD25^+^ Tregs (Fig. 4g and Supplementary Table 2). The commonly upregulated gene enrichment identified in both CD103^+^ and CD25^+^ Tregs in RT + Conc.IT-treated glioblastoma included immunosuppression pathways related to kynurenine and tryptophan metabolism^55^ (Fig. 4h and Supplementary Table 9). Analyses of the differences underlining CD103^+^ and CD25^+^ Treg immune activation revealed that RT + Conc.IT CD25^+^ Tregs displayed higher *Klrg1* and Th2-associated cytokine (*Il-4* and *Il-5*) expression (Extended Data Fig. 6b and Supplementary Tables 10 and 12). RT + Conc.IT CD103^+^ Tregs presented increased *Flt3* expression and high levels of genes and pathways involved in PPAR signaling and cholesterol and lipid metabolism (Fig. 4i,j, Extended Data Fig. 6b and Supplementary Tables 11 and 12). Interestingly, glycolytic metabolic pathways support Treg proliferation and inflammatory functions^56,57^, while lipid signaling and anabolic metabolism regulate the functionally suppressive state of Tregs in the TME^58,59^.

We further explored the functional differences between CD103^+^ and CD25^+^ Treg populations by performing ex vivo suppression assays^60^ of CD8^+^ T cells co-cultured with CD25^−^ T cells, CD25^+^ or CD103^+^ Tregs isolated from d12 RT + Conc.IT-treated glioblastoma (Extended Data Fig. 6a). Flow cytometry analysis revealed that RT + Conc.IT CD103^+^ Tregs were substantially more immunosuppressive than CD25^+^ Tregs, with CD8^+^ T cells acquiring a more exhausted (CD39), and significantly less proliferative (Ki67) and cytotoxic (IFNy, GrzA and GrzB) phenotype when co-cultured with CD103^+^ Tregs (Fig. 4k). Interestingly, CD103^+^ Treg suppressive capacity proved to be tumor specific, as no differences in CD8^+^ T cell profiles were found when co-cultured with Tregs sorted from spleens of RT + Conc.IT-treated mice (Extended Data Fig. 6c).

Altogether, these results indicate that RT + Conc.IT treatment not only increases glioblastoma Treg content, but enhances specific subset immunosuppressive capacities, potentially through metabolic pathway alterations, thereby suppressing Teff cell cytotoxic activity.

### Treg depletion results in TLS formation

**Fig. 5:**
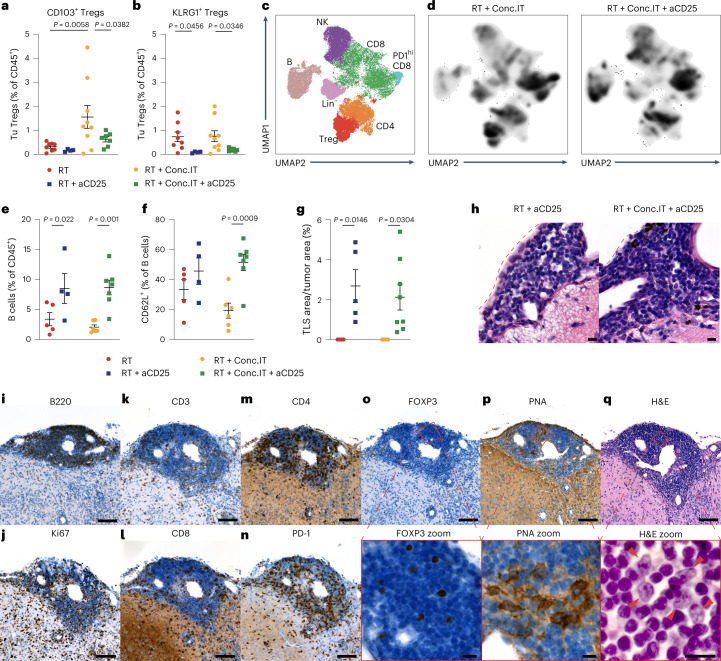
Targeting CD25^+^ regulatory T cells results in the formation of TLS in glioblastoma. **a**,**b**, Flow cytometry quantification of CD103^+^ Tregs (**a**) and KLRG1^+^ (**b**) Tregs in PDG-Ink4a tumors (Tu, tumor; for treatment schedule, see Extended Data Fig. 7a). RT and RT + Conc.IT data points are from Fig. 4d supplemented with three additional data points per treatment group (RT *n* = 8, RT + aCD25 *n* = 4, RT + Conc.IT *n* = 9, RT + Conc.IT + aCD25 *n* = 7 mice). **c**,**d**, UMAP projections and unsupervised FlowSOM clustering analysis of CD45^+^CD11b^−^ cells isolated from PDG-Ink4a tumors identified seven main populations: B, B cells; NK, NK cells; CD8, CD8^+^ T cells; PD-1^hi^CD8, CD8^+^ T cells with high PD-1 expression; Treg, regulatory T cells; CD4, CD4^+^ T cells; Lin^−^, cells negative for lineage markers (RT *n* = 5, RT + aCD25 *n* = 4, RT + Conc.IT *n* = 6, RT + Conc.IT + aCD25 *n* = 7 mice). **d**, Density projection plots from **c** of RT + Conc.IT and RT + Conc.IT + aCD25 treatment groups. **e**,**f**, Flow cytometry quantification of CD19^+^ B cells (**e**) (gated from CD45^+^CD11b^+^CD3) and CD62L^+^ cells (**f**) (% of CD19^+^ B cells from **e**; RT *n* = 8, RT + aCD25 *n* = 4, RT + Conc.IT *n* = 8, RT + Conc.IT + aCD25 *n* = 7 mice). **g**, Quantification of TLS area as a percentage of total tumor area in the different treatment groups (RT *n* = 4, RT + aCD25 *n* = 5, RT + Conc.IT *n* = 4, RT + Conc.IT + aCD25 *n* = 8 mice). **h**, Representative H&E staining of TLS quantified in **g** (scale bars, 10 µm; representative of *n* = 8 independent repeats). **i**–**q**, Representative image of a TLS in RT + Conc.IT + aCD25-treated PDG-Ink4a tumor sequentially stained for B220 (**i**), Ki67 (**j**), CD3 (**k**), CD8 (**l**), CD4 (**m**), PD-1 (**n**), FOXP3 (**o**), PNA (**p**) and H&E (**q**). Red squares (**o**–**q**) indicate magnified areas. Red arrows (**q**) identify lymphoblastic-like cells within the TLS. Scale bars, 100 µm for the main and 10 µm for the magnified panels (**i**–**q**). Representative of *n* = 8 independent repeats. For all graphs, analyses were done at d12 post treatment initiation on the tumor-containing brain quadrant. Statistics: one-way ANOVA with Benjamini, Krieger and Yekutieli correction for multiple testing (**a**, **b** and **e**–**g**). Data are represented as mean ± s.e.m. (**a**, **b** and **e**–**g**). Source data

**Extended Data Fig. 7:**
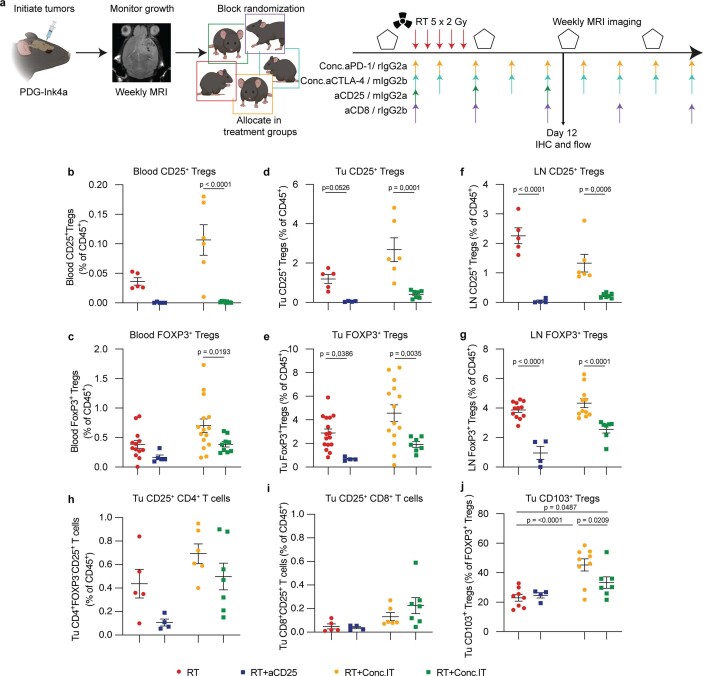
Alterations of the systemic and local immune cell contexture post CD25-mediated depletion of Tregs in glioblastoma combination treatment. **a**, Schematic overview of the experimental design. PDG-Ink4a tumors were initiated as described in Methods. At 4-7 weeks post tumor initiation, tumor size was quantified by MRI. Based on tumor volume, mice were distributed into treatment groups with (anti-CD25 (aCD25), anti-CTLA-4 (aCTLA-4), RT, RT combined with anti-CD25 (RT + aCD25), RT + Conc.IT, RT + Conc.IT combined with anti-CD25 (RT + Conc.IT + aCD25), RT + Conc.IT + aCD25 combined with anti-CD8 (RT + Conc.IT + aCD25 + aCD8), RT combined with concurrent anti-CTLA-4 immunotherapy (RT + aCTLA-4), RT + aCTLA-4 combined with anti-CD25 (RT + aCTLA-4 + aCD25). Anti-PD-1 and anti-CTLA-4 treatment were administered every third day until endpoint, anti-CD25 at 0d, 5d and 11d and anti-CD8 treatment every sixth day until endpoint. Mice were followed-up weekly by MRI and sacrificed for immunohistochemical and flow cytometry analysis at d12 post treatment initiation or for survival analysis at humane endpoint or at experimental endpoint (80d). The schematic was created using BioRender.com. **b**^**−**^**g**, Flow cytometry quantification of CD25^+^ Tregs (gated from CD45^+^CD11b^−^CD3^+^CD4^+^FOXP3^+^ T cells), FOXP3^+^ Tregs (gated from CD45^+^CD11b^-^CD3^+^CD4^+^ T cells), CD25^+^ CD4^+^FOXP3^−^ T cells (gated from CD45^+^CD11b^−^CD3^+^CD4^+^FOXP3^−^ T cells) and CD25^+^ CD8^+^ T cells (gated from CD45^+^CD11b^−^CD3^+^ T cells) of PDG-Ink4a tumor-bearing mice. **b,c**, CD25^+^ Tregs (**b**) and FOXP3^+^ Tregs (**c**) in the blood at d6-7 after treatment start (**b**: RT n = 5, RT + aCD25 n = 5, RT + Conc.IT n = 6, RT + Conc.IT + aCD25 n = 9 mice. **c**: RT n = 13, RT + aCD25 n = 5, RT + Conc.IT n = 15, RT + Conc.IT + aCD25 n = 9 mice). **d,e**, Intratumoral CD25^+^ Tregs (**d**) and FOXP3^+^ Tregs (**e**) at d12 (**d**: RT n = 5, RT + aCD25 n = 4, RT + Conc.IT n = 6, RT + Conc.IT + aCD25 n = 7 mice. **e**: RT n = 16, RT + aCD25 n = 4, RT + Conc.IT n = 14, RT + Conc.IT + aCD25 n = 7 mice). **f,g**, CD25^+^ Tregs (**f**) and FOXP3^+^ Tregs (**g**) in the superficial cervical LN at d12 (**f**: RT n = 5, RT + aCD25 n = 4, RT + Conc.IT n = 6, RT + Conc.IT + aCD25 n = 7 mice. **g**: RT n = 12, RT + aCD25 n = 4, RT + Conc.IT n = 11, RT + Conc.IT + aCD25 n = 7 mice). **h,i**, Flow cytometry quantification of intratumoral CD25^+^ CD4^+^FOXP3^−^ T cells (**h**) and CD25^+^ CD8^+^ T cells (**i**) at d12 (**h**: RT n = 5, RT + aCD25 n = 4, RT + Conc.IT n = 6, RT + Conc.IT + aCD25 n = 7 mice. **i**: RT n = 5, RT + aCD25 n = 4, RT + Conc.IT n = 6, RT + Conc.IT + aCD25 n = 7 mice). **j**, Flow cytometry quantification of intratumoral CD103^+^ Tregs at d12 (RT n = 8, RT + aCD25 n = 4, RT + Conc.IT n = 9, RT + Conc.IT + aCD25 n = 7 mice). For (**c,e,g**), RT and RT + Conc.IT data points include data from Fig. 3n (**e**) and Fig. 5b,c (**c,g**). Statistics: one-way ANOVA with Benjamini, Krieger and Yekutieli correction for multiple testing (**b-j**). Data are represented as mean ± S.E.M. (**b-j**). Source data

To address the functional role of Tregs in restraining RT + IT efficacy, we opted to implement treatment with a pan-Treg-targeting CD25-depleting antibody (aCD25) (ref. ^52^) during the response phase of Treg induction in RT + Conc.IT-treated PDG-Ink4a glioblastoma-bearing mice (Extended Data Fig. 7a). Tregs were efficiently depleted in the systemic circulation (Extended Data Fig. 7b,c), the glioblastoma TME (Extended Data Fig. 7d,e) and LN compartment (Extended Data Fig. 7f,g). Notably, CD4^+^ and CD8^+^ T cells displayed minimal CD25 expression, and were not significantly reduced upon CD25-targeted depletion (Extended Data Fig. 7h,i). Importantly, aCD25 treatment targeted CD103^+^ and KLRG1^+^ Tregs, as both subsets were efficiently depleted in the TME at d12 (Fig. 5a,b). However, the remaining FOXP3^+^CD103^+^ Tregs still present in the TME following CD25 depletion (Extended Data Fig. 7j) suggest that a portion of these cells are CD25^−^ and cannot be depleted using this strategy.

**Extended Data Fig. 8:**
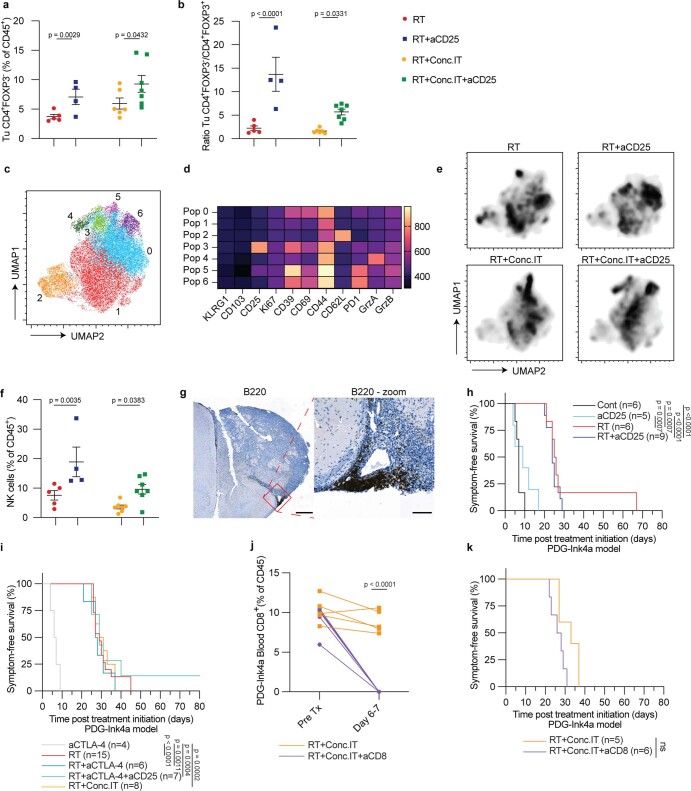
CD25-mediated Treg depletion combined with RT + Conc.IT leads to TLS formation, increased effector T cell levels and a survival benefit. **a,b**, Flow cytometry quantification of CD4^+^FOXP3^−^ T cells (gated from CD45^+^CD11b^−^CD3^+^ T cells) (**a**) and of CD4^+^FOXP3^−^ T cells/CD4^+^FOXP3^+^ Treg ratio (gated from CD45^+^CD11b^−^CD3^+^ T cells) (**b**). For **a,b**, RT n = 5, RT + aCD25 n = 4, RT + Conc.IT n = 6, RT + Conc.IT + aCD25 n = 7 mice. **c**, UMAP projection and unsupervised FlowSOM clustering of the CD4^+^ T cell population (from Fig. 5c) identified 7 distinct subpopulations of CD4^+^ T cells (Pop 0-6). **D**, Heatmap depicting the MFI of activation markers in the CD4^+^ T cell subpopulations identified in (**c**). **e**, UMAP density projections plot of CD4^+^ T cell subpopulations identified in RT, RT + aCD25, RT + Conc.IT and RT + Conc.IT + aCD25 treatment groups. For **c-e**, RT n = 5, RT + aCD25 n = 4, RT + Conc.IT n = 6, RT + Conc.IT + aCD25 n = 7 mice. **f**, Flow cytometry quantification of NK1.1^+^ NK cells (gated from CD45^+^CD11b^−^CD19^−^CD3^−^; RT n = 5, RT + aCD25 n = 4, RT + Conc.IT n = 7, RT + Conc.IT + aCD25 n = 7 mice). **g**, Representative image of immunohistochemical staining for B220 in RT + Conc.IT + aCD25 treated tumors (scale bar: 500 um for main panel, 50 um for magnified panel; representative of n = 8 independent repeats). **h**, Kaplan–Meier survival curve of PDG-Ink4a tumor-bearing mice treated with Cont, aCD25, RT or RT + aCD25 (see Extended Data Fig. 7a for treatment schedule). **i**, Kaplan–Meier survival curve of PDG-Ink4a tumor-bearing mice treated with aCTLA-4, RT, RT + aCTLA-4, RT + Conc.IT (aPD-1) or RT + aCTLA-4 + aCD25 (see Extended Data Fig. 7a for treatment schedule). **j**, Flow cytometry quantification of CD8^+^ T cells (gated from CD45^+^CD11b^−^CD3^+^ cells) in the blood of PDG-Ink4a glioblastoma-bearing mice at the indicated treatment groups. Each line indicates the matched quantification before start of treatment and at d6-7. **k**, Kaplan–Meier survival curves of PDG-Ink4a tumor-bearing mice treated with RT + Conc.IT or RT + Conc.IT + aCD8 (RT + Conc.IT n = 5 and RT + Conc.IT + aCD8 n = 6 mice). For **a-g**, analyses were done at d12 post treatment initiation. Statistics: one-way ANOVA with Benjamini, Krieger and Yekutieli correction for multiple testing (**a,b,f**), log-rank test (**h,i,k**) and two-sided unpaired t-test (**j**). Data are represented as mean ± S.E.M (**a,b,f**). Mice depicted in the survival curves were treated within the same cohorts (**h,i,k**). Median survival and significance depicted in Supplementary Table S1 (**h,i,k**). Source data

Next, we explored the changes in the immune microenvironment caused by CD25^+^ Treg depletion. UMAP and FlowSOM clustering analyses of lymphoid cells independently confirmed Treg depletion in response to RT + Conc.IT + aCD25 treatment (Fig. 5c,d). Moreover, Treg depletion resulted in an increased CD4^+^ T cell population independently of IT addition (Extended Data Fig. 8a,b). UMAP and FlowSOM clustering analyses of conventional CD4^+^ T cells identified seven subpopulations with distinct abundance across RT-, RT + aCD25-, RT + Conc.IT- and RT + Conc.IT + aCD25-treated tumors (Extended Data Fig. 8c–e). CD25 depletion led to increased activated CD4^+^ T cell content (GrzA^+^CD44^high^CD62^low^; population 4), while an exhausted subpopulation (CD39^int^CD44^int^CD62^low^; population 0) was abundant in RT + Conc.IT-treated tumors (Extended Data Fig. 8d,e). Interestingly, Treg depletion negatively affected the presence of an activated but exhausted CD4^+^ T cell subset (population 5—CD39^high^CD44^high^CD62^low^) while a comparable but less exhausted CD39^int^CD44^high^CD62^low^ subset (population 6) was acquired in RT + Conc.IT + aCD25-treated tumors (Extended Data Fig. 8d,e). Altogether, these findings suggest that conventional CD4^+^ T cells participate in RT + Conc.IT + aCD25 treatment efficacy, potentially by inducing a more mature and activated CD4^+^ Teff cell pool upon Treg depletion.

CD25^+^ Treg depletion also induced an increased B and NK cell abundance, independent of IT treatment (Fig. 5d,e and Extended Data Fig. 8f). However, within the B cell fraction, CD25 depletion in RT + Conc.IT tumors specifically increased CD62L^+^ cells within an immature phenotype, whereas this was not apparent in response to RT + aCD25 treatment (Fig. 5f). We hypothesized that this may be indicative of lymphoid neogenesis, given the recent identification of meningeal TLS in patients with glioblastoma^19^. Indeed, immunohistochemical analyses identified TLS presence in d12 RT + aCD25- and RT + Conc.IT + aCD25-treated tumors (Fig. 5g). Pathological assessment asserted lymphoid aggregate formation containing B cells in close association with the meninges in aCD25-treated mice (Fig. 5h and Extended Data Fig. 8g). Immunohistochemical staining identified these clusters to consist of Ki67^+^ proliferative cells, B cells, Tregs, CD8^+^ T and CD4^+^ T cells (Fig. 5i–o), confirming the increases previously shown with FlowSOM and UMAP analysis (Fig. 5c,d and Extended Data Fig. 8a–e). TLS were highly infiltrated by PD-1^+^ cells, indicating that their immunomodulatory role may be altered by α-PD-1 treatment (Fig. 5n). Regardless of ICB administration, TLS contained structures expressing the germinal center marker peanut agglutinin (PNA) (Fig. 5p) and lymphoblast-like cells (Fig. 5q), which both indicate ongoing naive T cell differentiation within these structures. Thus, aCD25-targeted Treg depletion results in TLS induction in which active antigen presentation takes place, suggesting that Tregs hamper local presentation of antigens in TLS. Our results suggest that, when enforced in the TME, TLS might participate to heighten Teff and cytotoxic T cell responses in the context of ICB therapy, thereby not acting as predictive biomarkers of clinical outcome, but as structures correlated with immunotherapeutic responses in the central nervous system.

### Treg targeting improves survival in response to RT

**Fig. 6:**
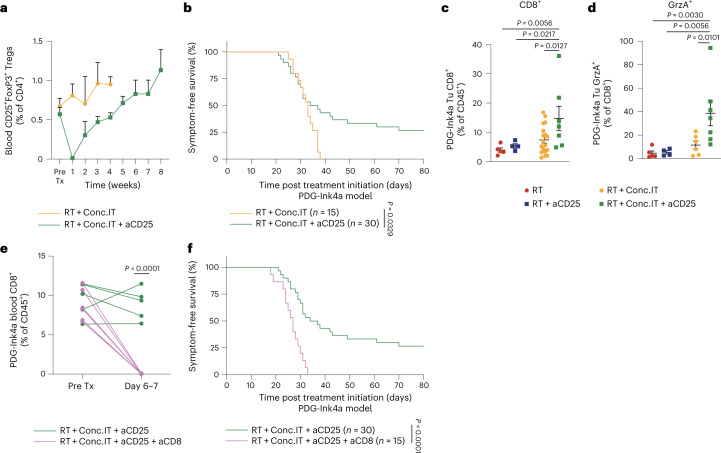
Combination treatment of RT and CD25^+^ Treg targeting improves survival in a CD8^+^ T cell-dependent manner. **a**, Flow cytometry quantification of CD25^+^ Tregs (gated from CD45^+^CD11b^−^CD3^+^CD4^+^FOXP3^+^ T cells) in the blood of PDG-Ink4a tumor-bearing mice treated with RT + Conc.IT or RT + Conc.IT + aCD25 (Pre Tx, before treatment; RT + Conc.IT *n* = 6, RT + Conc.IT + aCD25 *n* = 11 mice). **b**, Kaplan–Meier survival curves of PDG-Ink4a tumor-bearing mice treated with 5x2Gy RT + Conc.IT or RT + Conc.IT + aCD25 (for treatment schedule, see Extended Data Fig. 7a). **c**, Flow cytometry quantification of CD8^+^ T cells (gated from CD45^+^CD11b^−^CD3^+^ cells) in d12-treated PDG-Ink4a glioblastoma (RT *n* = 5, RT + aCD25 *n* = 4, RT + Conc.IT *n* = 18, RT + Conc.IT + aCD25 *n* = 7 mice). **d**, Flow cytometry quantification of GrzA^+^ CD8^+^ T cells from **c** (RT *n* = 5, RT + aCD25 *n* = 4, RT + Conc.IT *n* = 6, RT + Conc.IT + aCD25 *n* = 7 mice). **e**, Flow cytometry quantification of CD8^+^ T cells (gated from CD45^+^CD11b^−^CD3^+^ cells) in the blood of PDG-Ink4a glioblastoma-bearing mice from the indicated treatment groups. Each line indicates the matched quantification before start treatment and at d6–7 (RT + Conc.IT + aCD25 *n* = 5, RT + Conc.IT + aCD25 + aCD8 *n* = 6 mice). **f**, Kaplan–Meier survival curves of PDG-Ink4a tumor-bearing mice treated with RT + Conc.IT + aCD25 or RT + Conc.IT + aCD25 + aCD8. Statistics: log-rank test (**b** and **f**), one-way ANOVA with Benjamini, Krieger and Yekutieli correction for multiple testing (**c** and **d**), two-tailed unpaired *t*-test (**e**). Data are represented as mean + s.e.m. (**a**) or ± s.e.m. (**c** and **d**). Median survival and significance depicted in Supplementary Table 1 (**b** and **f**). Source data

Having established that CD25-depleting antibodies shape the TME immune composition during the early response phase to RT + IT, we next assessed the long-term effects of Treg targeting in the PDG-Ink4a model (Extended Data Fig. 7a). Although aCD25 effectively depleted Tregs (Extended Data Fig. 7b–g), this effect was transient, and circulating CD25^+^ Treg content subsequently increased over time (Fig. 6a). We next assessed the therapeutic response to CD25 depletion and observed that RT + aCD25-treated PDG-Ink4a glioblastoma-bearing mice did not experience any survival benefit over RT monotherapy (Extended Data Fig. 8h and Supplementary Table 10). However, RT + IT + aCD25 treatment led to long-term survival benefit with complete tumor control in a subset of mice (Fig. 6b and Supplementary Table 1). Interestingly, this effect seemed α-PD-1 ICB specific, as RT + aCTLA-4 + aCD25 treatment did not induce such a survival benefit (Extended Data Fig. 8i and Supplementary Table 1). These results reveal a window of opportunity provided by short-term Treg depletion to unleash α-PD-1 efficacy specifically, which may be translationally relevant to patients with glioblastoma.

As CD103^+^ Tregs are potent suppressors of CD8^+^ T cell responses (Fig. 4k), we next examined both CD8^+^ T cell content and their activation state in RT + Conc.IT + aCD25-treated glioblastoma and observed an accumulation of CD8^+^ T cells with elevated GrzA expression (Fig. 6c,d), confirming the results obtained in ex vivo Treg suppression assays. Subsequently, we investigated whether CD8^+^ T cells were central mediators of the RT + Conc.IT + aCD25-conferred survival benefit (Fig. 6b and Supplementary Table 1). Following treatment with an aCD8 targeting antibody administered before RT + IT (Extended Data Fig. 7a), CD8^+^ T cells were effectively depleted in the systemic circulation (Fig. 6e and Extended Data Fig. 8j). Strikingly, the survival benefit observed with RT + Conc.IT + aCD25 was lost by co-targeting CD8^+^ T cells in long-term preclinical trials of PDG-Ink4a glioblastoma-bearing mice (Fig. 6f, Extended Data Figs. 7 and 8k and Supplementary Table 1). We therefore conclude that RT + IT combined with aCD25-Treg targeting enhances glioblastoma survival in a CD8^+^ T cell-dependent manner.

## Discussion

The lack of α-PD-1 ICB therapeutic efficacy in phase III clinical trials of patients with glioblastoma^3^ (CheckMate 498 and CheckMate 548) underscores the need for multidimensional targeting of the complex immunosuppressive milieu of primary brain tumors, to achieve therapeutic efficacy in this disease of high clinical unmet need. We analyzed the glioblastoma TME and its evolution in response to combined standard-of-care and ICB therapy, and demonstrate that Tregs are strikingly affected by RT + IT and impair its therapeutic efficacy.

Although a low TMB has been associated with decreased ICB response in a wide range of solid tumors^9^, it has recently been correlated with a more inflamed phenotype and improved immunotherapeutic response in glioblastoma^13^, while patients with hypermutated glioblastoma showed low response rates to ICB^12^. By generating an exogenous antigen expressing glioblastoma GEMM that recapitulates the pathology, invasiveness and TME of lowly immunogenic models and patients with glioblastoma, we demonstrated that enforced immunogenic antigen expression does not enable IT efficacy in glioblastoma. Rather, the observed ICB therapeutic responses in brain metastases^6^ and immune-reactive, preclinical glioblastoma models may be mediated by immune contexture differences, including a lack of microglia dominance and notable T cell-rich TME, compared with the lymphoid-deserted primary glioblastoma landscape. Indeed, analyses of matched primary and recurrent glioblastoma patient samples confirmed that relapsed tumors display increased T cell infiltration^14^, which, in addition to the reported tumor-intrinsic effects, may participate in neoadjuvant ICB efficacy^4,18^ independent of changes in antigen availability or TMB.

Currently, most ICB phase III trials in primary glioblastoma have employed RT + Conc.IT α-PD-1 administration (CheckMate 498 and CheckMate 548), although a mechanistic rationale for this therapeutic scheduling was lacking. Hence, therapeutic response may have been undermined by immunosuppressive feedback pathways in the course of combination treatment, as suggested by the prolonged Treg induction with altered metabolic features we identified in RT + Conc.IT-treated glioblastoma. Importantly, RT + Adj.IT but not RT + Conc.IT resulted in a modest, albeit significant survival benefit, with increased proinflammatory cytokine signaling in abundantly present CD4^+^ conventional T cell subsets. We therefore propose that RT + Adj.IT administration is superior to RT + Conc.IT schedules used in phase III clinical trials, potentially by maximally exploiting the induction of intratumoral T cells post RT and hindering immunosuppressive feedbacks in the TME.

Regardless of therapeutic IT scheduling, Tregs are induced by ICB in both the TME and LN. The long-term survival benefit effects conveyed by combinatorial RT, α-PD-1 and aCD25 targeting were specific to this ICB treatment, as no therapeutic benefit was achieved with α-CTLA-4, confirming Treg therapeutic relevance in improving α-PD-1 response in a CD8^+^ T cell-dependent manner. While Tregs have previously been involved in α-PD-1 response^57^, it remained unclear whether enhanced Treg response was mediated by local differentiation or systemic expansion and recruitment of Tregs. As the circulating Treg content is unchanged and Tregs proliferate within the tumor, we propose that both local expansion and CD4^+^ conventional T cell conversion act in concert to increase Treg content in RT + Conc.IT-treated glioblastoma. While α-PD-1 combined with GITR reprogramming of CD4^+^ T cells has been reported to improve ICB response in glioblastoma^24^, this is the first time the dynamic response of intratumoral Teff and Treg subsets to RT and RT + ICB is described and timely harnessed in poorly immunogenic glioblastoma. Indeed, our results revealed that phenotypic changes in α-PD-1-treated intratumoral Tregs occur in addition to subset enrichment, with an increased CD103^+^ Treg subpopulation displaying elevated cholesterol and lipid metabolism pathways, previously identified as immunosuppressive Treg features^58^.

Therapeutic Treg depletion has been employed in clinical trials, and a first-generation aCD25 antibody showed tolerable toxicity in patients with glioblastoma^61,62^. Recently, a phase I dose-escalation trial of a third-generation aCD25 antibody, which efficiently depleted Tregs while maintaining IL-2 signaling activity^53^, was launched (NCT04158583) and a follow-up phase Ib study with combined PD-L1 ICB has been initiated for a range of solid tumors. These advances underscore that Treg targeting approaches combined with ICB may represent viable treatment strategies for patients with glioblastoma. A comprehensive understanding of not only the immune cell phenotype at baseline but also their adaptive response to therapy is critical to evaluate ongoing clinical trial successes. Indeed, one possible consequence of using aCD25 neutralizing antibodies may be that not all Tregs are targeted, as suggested by our results on the subset of CD103^+^ Tregs lacking CD25 expression that may be functionally important in therapy resistance mechanisms. Importantly, the genetic drivers of glioblastoma malignancy will probably affect the successful translational applicability of Treg targeting, as we observed a less pronounced Treg induction and response in the genetically distinct PDG-Ink4a and PDG-p53 glioblastoma models. In light of this and other studies^58^ suggesting that metabolic adaptation can enforce Treg functional specialization, further work is needed to establish the molecular mechanisms underlying the effects of metabolic changes on the tumor Treg pool. Consequently, additional avenues to reprogram T cells could be considered aside from CD25 targeting, from metabolic rewiring with diet intervention to blocking lipid metabolism.

Our study demonstrates that the rational design of therapeutic regimens boosting immune sensitization of the glioblastoma microenvironment is needed to overcome immunosuppression and achieve therapeutic benefit. While neoadjuvant ICB administration in recurrent glioblastoma may capitalize on increased T cell infiltration and cDC1 activation^4,18^, our results exposed a therapeutic window post-RT for primary glioblastoma treatment. Combined RT and Treg targeting sensitizes an otherwise lymphoid-scarce glioblastoma into more inflamed tumors with >tenfold intratumoral T cell infiltration and meningeal TLS formation. The historic view considering glioblastoma as an immune desert is being revisited^63^, especially with the recent identification of brain lymphatics^64,65^ and TLS^19,66^. Our study suggests that TLS induction ensues Treg targeting independently of ICB treatment and unleashes CD8^+^ T cell responses, bearing promising potential for different immune cell-based therapeutic approaches^21,67–69^. Altogether, these findings provide a framework for the design of T cell-centric immunotherapies in glioblastoma and warrant investigation of ICB, TLS and Treg targeting in a clinical setting.

## Methods

This research complies with all relevant ethical regulations of the Netherlands Cancer Insitute/Antoni van Leewenhoek and McGill Cancer Center with the Animal Welfare Committee and NKI-biobank CFMPB541 approval.

### Glioblastoma mouse model generation

Nestin-Tv-a;*Ink4a/Arf*^−*/*−^ mice (BL/6 background) and Nestin-Tv-a mice (BL/6 background) have been previously described and were bred within the Netherlands Cancer Institute (NKI) animal facility. The RCAS-PDGFB-driven mouse models of gliomagenesis (PDG) have been previously described^16,26,27,70–72^. Briefly, glioblastomas were induced in 5–6-week-old male and female mice by intracranial injection of DF-1 cells expressing an RCAS vector encoding PDGF-B HA in *Nestin-Tv-a*;*Ink4a/Arf*^−*/*−^ mice (PDG-Ink4a model), or DF-1 cells expressing PDGF-B HA and a short hairpin RNA targeting *TP53* in *Nestin-Tv-a* mice (PDG-p53).

The PDG-Ink4a-OVA model was developed by cloning the OVA sequence into the RCASBP-Y vector. DF1 cells were transfected using the calcium phosphate transfection kit (ThermoFisher) to generate DF1-OVA cells. Successful transfection was confirmed by flow cytometry assessment of OVA expression (Extended Data Fig. 2a). To induce tumor development, *Nestin-Tv-a*;*Ink4a/Arf*^*-/-*^ mice were intracranially injected with a 1:1 ratio of 200,000 DF1-PDGFB and 200,000 DF1-OVA cells. For the GL261 model, 20,000 GL261 cells were intracranially injected in C57BL/6JRj mice (Janvier labs) to induce tumor development. All animal studies were approved by the Institutional Animal Care and Use Committees of the NKI.

### Preclinical in vivo studies

All mouse procedures were approved by the animal ethics committee of the NKI and performed in accordance with institutional, national and European guidelines for animal care and use. Magnetic resonance imaging (MRI) scans were performed weekly to monitor tumor development. Mice were distributed into treatment groups by block randomization at a tumor volume >20 mm^3^ and <90 mm^3^ (PDG GEMMs) or ~10 mm^3^ (GL261). RT was performed after sedation by isoflurane, and irradiation of the tumor-containing quadrant was performed using a X-RAD 320 or X-RAD SmART (Precision X-Ray) five times daily at 2 Gy doses each. α-PD-1 (BioXCell) was administered every third day until endpoint at 200 µg per dose. α-PD-1 treatment was initiated before the first dose of RT for the concurrent treatment schedule and 1 day after the last dose of RT for the adjuvant treatment schedule. Treg depletion was performed by administration of 200 µg of anti-CD25 (developed by S. Quesada and obtained through Evitria) on days 0, 5 and 11. CD8^−^ depletion (BioXCell) was performed by administration of 400 µg anti-CD8 at day 0, followed by 100 µg maintenance doses every 6 days until endpoint. rIgG2a (for α-PD-1), mIgG2a (for anti-CD25) and rIgG2a (for anti-CD8) were administered as isotype controls in equivalent timing and dosage as the treatment antibodies. All in vivo antibodies and used dilutions are listed in Supplementary Table 13. Animals were killed at specified timepoints or upon recurrence of the tumor as monitored by regular MRI imaging, or by neurological symptoms, as approved by the Institutional Animal Care and Use Committee of the NKI.

### Institutional review board approval and patient information

Human specimens were obtained through the NKI-biobank CFMPB541 with patient consent. Data on patients diagnosed with confirmed grade IV glioma and no prior history of brain malignancy were collected after surgical resection (primary tumors). The same patients underwent fractionated RT and temozolomide as part of the standard-of-care and recurrent disease resection were collected in matched patients (recurrent) and used for paired analyses.

### Cell culture

DF1 chicken fibroblasts were obtained from the American Type Culture Collection and were cultured in Dulbecco’s modified Eagle medium (DMEM; Life Technologies) supplemented with 4.5 g d-glucose, 110 mg l^−1^ sodium pyruvate, 10% (vol/vol) heat-inactivated fetal bovine serum and 1% (vol/vol) penicillin/streptomycin (P/S). RCAS vectors expressing Platelet-Derived Growth Factor β-hemagglutinin (PDGFB-HA), and a short hairpin against mouse TP53 (shP53) were provided by T. Ozawa and E. Holland (Fred Hutchinson Cancer Research Center)^29^. GL261 cells were provided as a kind gift from J. Joyce lab and were cultured in DMEM supplemented with 4.5 g d-glucose, 110 mg l^−1^ sodium pyruvate, 10% (vol/vol) heat-inactivated fetal calf serum and 1% (vol/vol) penicillin/streptomycin . All cell lines were cultured at 37 °C and 5% CO_2_ in a humidified cell incubator. The cell lines were not authenticated after purchase but routinely tested negative for mycoplasma contamination (Lonza).

### Treg suppression assay

The Treg-CD8^+^ T cell suppression assay was previously described^60^. Briefly, CD25^−^ T cells (CD45^+^CD11b^−^CD3^+^CD8^−^CD4^+^KLRG1^−^), CD25^+^ Tregs (CD45^+^CD11b^−^CD3^+^CD8^−^CD4^+^) and CD103^+^ Tregs (CD45^+^CD11b^−^CD3^+^CD8^−^CD4^+^KLRG1^−^) were sorted from freshly isolated tumors and spleen of glioblastoma-bearing mice 12 days after RT + Conc.IT initiation, and activated overnight in a 96-well plate with Iscove’s modified Dulbecco’s medium containing 8% fetal calf serum, 100 IU ml^−1^ penicillin, 100 µg ml^−1^ streptomycin, 0.5% β-mercapto-ethanol, 300 U ml^−1^ IL-2, 1:5 bead:cell ratio CD3/CD28 coated beads (ThermoFisher). Responder CD8^+^ T cells (CD45^+^CD11b^−^CD3^+^) were rested overnight in a 24-well plate. After 24 h, responder cells were mono- or co-cultured with CD25^−^ T cells, CD25^+^ Tregs or CD103^+^ Tregs, supplemented with CD3/CD28 beads (1:5 bead:cell ratio) for 24 h, without additional IL-2. Cells were then collected and stained for 24-color spectral flow cytometric analysis (Supplementary Table 13).

### Flow cytometry and cell sorting

Tissues were collected in ice-cold phosphate-buffered saline (PBS), and blood was collected in heparin-containing tubes. Tumors were macroscopically dissected and all nontumor brain tissue was removed, unless otherwise stated in the figure legends. Blood samples were collected in potassium/EDTA-coated tubes, and erylysis was performed for 10 min using lysis buffer (8.4 g NH_4_Cl + 1.2 g NaHCO_3_ + 0.2 ml 0.5 M EDTA in 1 litre PBS). Superficial cervical LNs were digested by 3 mg ml^−1^ collagenase type A (Roche) and 25 µg ml^−1^ DNase (Sigma) in serum-free DMEM medium for 20 min at 37 °C. Single-cell suspensions of brain tumors were obtained by enzymatic dissociation using a gentleMACS Octo Dissociator and the Tumor Dissociation kit (Miltenyi Biotec). Tumor and LN cell suspensions were subsequently passed through a 40 μm strainer (Corning, Sigma-Aldrich). Myelin depletion was then performed on tumor samples using Myelin Removal Beads II on MS columns (Miltenyi Biotec). Single-cell suspensions were then subjected to Fc receptor blocking (rat anti-mouse CD16/32, BD Biosciences) for 15 min at 4 °C and stained with conjugated antibodies for 30 min at 4 °C in the dark in 2% fetal calf serum in PBS. Zombie NIR or Zombie Aqua (BioLegend) staining was performed to discriminate live and dead cells followed by fixation and permeabilization using the Cytofix/Cytoperm kit (BD Biosciences) to stain for intracellular proteins. All antibodies and used dilutions are listed in Supplementary Table 13. Samples were acquired using a BD LSRFortessas (BD BioSciences) or a Cytek Aurora (Cytek), and cells were sorted using a FACSAria Fusion (BD BioSciences). Data analysis including quantification and data visualization were performed using FlowJo Software version 10.7.1 (BD BioSciences) and GraphPad Prism 9.0.0 (GraphPad software).

For multidimensional data visualization and analyses, data obtained from a 24-color (Figs. 4 and 5 and Extended Data Fig. 8) and 14-color (Extended Data Fig. 5) spectral flow cytometry panel were used. A total of 10,000 CD45^+^CD11b^+^, 2,240 CD4^+^ d6 and 1,139 CD4^+^ d12 live single cells per sample were downsampled using the the DownSample 3.3.1 plugin from the FlowJo Exchange. The FlowSOM 3.0.18, UMAP 3.1 and ClusterExplorer 1.6.3 plugins were then used to analyze immune populations from a concatenated dataset.

### WES

DNA was isolated from freshly frozen tissue biopsies of PDG-Ink4a, PDG-p53 and GL261 tumors or matched adjacent brain tissue using the DNeasy Blood & Tissue Kit (Qiagen). DNA was fragmented by Covaris shearing, after which the KAPA HTP DNA Library Kit (Roche) was used to prepare libraries. Exomes were enriched using SeqCap EZ MedExome probes (Roche), after which the libraries were sequenced with 150 basepair paired-end reads on the Novaseq SP (Illumina). To compare results with patient tumors, published WES data^30^ were analyzed in parallel. After adapter trimming using Seqpurge, sequences were aligned paired-end with Burrow–Wheeler aligner 0.7 using the MEM algorithm and duplicates were marked using Picard MarkDuplicates. Basecall quality recalibration was performed using GATK BaseRecalibrator, and single-nucleotide variants, insertions or deletions were called using GATK MuTect^73^. The resulting calls were annotated using SnpEff and Ensembl GRCm38.99. Nonsynonymous, exonic mutational load in coding genes was then determined by counting variants in the following classes: conservative and disruptive in-frame deletions, conservative and disruptive in-frame insertions, frameshift variant, missense variant, start lost, stop gained, stop lost, and stop-retained variant. Minimum coverage thresholds were >8-fold for patient brain samples and >16-fold for normal sample, while a minimum coverage of >2-fold tumor and >5-fold normal sample was used for mouse samples.

### Immunohistochemistry staining

At the indicated experimental endpoint or when mice reached their humane endpoint, mice were killed by carbon dioxide asphyxiation. Cardiac perfusion was performed with 10 ml PBS followed with 10 ml formalin. Tissues were then fixed in formalin for at least 2 days, and 2–3 mm-thick blocks were embedded in paraffin. Tissues were sectioned into 2–4-μm-thick slides and were deparaffinized by xylene and subsequently rehydrated. For histopathologic evaluation of tumor models, hematoxylin and eosin (H&E) staining was performed using a Tissue-Tek automated slide stainer, and slides were mounted with VECTASHIELD mounting medium (Vector). Histopathologic scoring was performed by a blinded independent pathologist (Ji-Ying Song). Quantification of TLS surface area was performed on H&E-stained slides using Qupath software version 0.2.3. For immunohistochemisty, rehydrated slides were subjected to Tris/EDTA antigen retrieval and endogenous peroxidases were inactivated with 3% H_2_O_2_ in methanol. After blocking in normal goat serum, sections were incubated with primary antibodies. All antibodies and used dilutions are listed in Supplementary Table 13. EnVision horseradish peroxidase-labeled polymer secondary antibodies (Agilent) were then used to enhance the signal that was visualized by incubation with 3,3′-diaminobenzidine. Slides were scanned using Pannoramic 1000 (3D Histech), and representative images were extracted using Slide Score (Slide Score).

### IMC—Hyperion

IMC was performed as described previously^74^. Briefly, antibodies were optimized and conjugated by the Single Cell and Imaging Mass Cytometry Platform at the Goodman Cancer Research Centre (McGill University) using Maxpar Conjugation Kits (Fluidigm). Formalin-fixed paraffin-embedded matched primary and recurrent glioblastoma samples were collected at the NKI-biobank (CFMPB541). Deparaffinization, heat-induced epitope retrieval with the Ventana Discovery Ultra auto-stainer platform (Roche Diagnostics), EZ Prep solution incubation (preformulated, Roche Diagnostics) and antigen retrieval in standard Cell Conditioning 1 solution (CC1, preformulated; Roche Diagnostics) were performed. After blocking in Dako Serum-free Protein Block solution (Agilent), antibody staining was performed overnight at 4 °C. Tissues were stained with a panel of 35 multiplexed metal-conjugated antibodies (Supplementary Table 13). IMC images were acquired at a resolution of roughly 1 μm, frequency of 200 Hz and area of 1 mm^2^, Hyperion Imaging System and CyTOF Software v.6.7.1014 (Fluidigm). Cell segmentation, intensity calculations, cell assignment and interaction/avoidance analyses were performed using a custom computational pipeline in MATLAB v.7.10. The foreground and background staining for each marker was modeled as a mixture of two Gaussians distributions. Cell segmentation was achieved by assessing the gradient magnitude, seed contour and scale space for each nucleus, followed by Chan-Vese80. Basic cell lineage assignments were defined by the following markers: cancer, PanCK^+^; macrophages, CD68^+^; neutrophils, MPO^+^; endothelial cells, CD31^+^; B cells, CD20^+^; cytotoxic T cells, CD3^+^CD8^+^ and helper T cells, CD3^+^CD4^+^. Histocat was used to generate representative images.

### RNA-seq analysis

FACS-isolated cell samples were sorted directly into RLT buffer (Qiagen), and preparation of RNA library and transcriptome sequencing was conducted by Novogene. For analyses, a raw count matrix was produced and loaded within the R environment (version 4.1.1). DESeq2 (version 3.14) was used to assess the differential gene expression between grouped samples using an absolute log_2_ fold change of 1 and a false discovery rate of 0.05. BioPlanet^75^, Jensen Compartments^76^, WikiPathways^77^, MsigDB^78^, Kyoto Encyclopedia of Genes and Genomes^79^ and Gene Ontology^37^ databases were used as a primary source for gene set overrepresentation analyses. Overrepresentation was assessed with the enrichR package^80^ to check whether an input set of genes significantly overlaps with annotated gene sets using a false discovery rate of 0.05. Gene set enrichment analysis was assessed with the GAGE package^81^, which uses the average of the absolute values of the per gene test statistics to account for both up- and downregulation of the curated pathways.

### Quantification and statistical analysis

Statistics were performed using GraphPad Prism 9.0.0. Statistical tests used are described in each of the panels of the figure legends. Data distribution is assumed to be normal, but this was not formally tested. For comparison of two-arm studies, two-sided unpaired *t*-tests were used as indicated. For comparison of multiple groups with a single variable and normally distributed continuous data, one-way analysis of variance (ANOVA) was used with Benjamini, Krieger and Yekutieli correction for multiple comparison. For comparison of multiple groups with two or more variables, two-way ANOVA was used with Benjamini, Krieger and Yekutieli method for multiple testing. RNA-seq pathway enrichment analysis was performed with two-sided Fisher’s exact test in combination with the Benjamini–Hochberg method for correction of multiple hypotheses testing, and differential gene expression between grouped samples was tested with a two-sided Wald test in combination with the Benjamini–Hochberg method for correction of multiple hypotheses testing. The Kaplan–Meier method with log-rank test was used for survival studies, of which the median survival and significance are depicted in Supplementary Table 1. Graphs show individual or in case of survival studies combined experiments/samples. Results are presented as mean with the error bars showing the standard error of the mean (s.e.m.) or standard deviation (s.d.). Differences with *P* < 0.05 were considered statistically significant.

For animal studies, sample size was determined with power calculation based on the mean and standard deviation from previous experimental results, and an alpha of 0.05 and power of 0.8 were taken as a guideline in these analyses. For ex vivo analysis, no statistical methods were used to predetermine sample sizes, but our sample sizes are similar to those reported in previous publications^16,27^. For all experiments, biological replicates were used to ensure reproducibility was ensured, with an *n* of at least 3.

For animal studies the block randomization method was used to prevent selection bias. Tumor volume measurement was performed blinded, but animal treatment was not, as strict treatment schedules had to be adhered to. Data analysis on collected and digested tissue was done blindly. Human data analyses were performed blinded by a third party.

### Reporting summary

Further information on research design is available in the Nature Portfolio Reporting Summary linked to this article.

## Supplementary information

Reporting Summary

Supplementary Tables Supplementary Tables 1–13.

## Data Availability

WES and RNA-seq data that support the findings of this study have been deposited in the Gene Expression Omnibus (GEO) under accession codes GSE203260. The dataset derived from this resource that supports the findings of this study is available at https://www.ncbi.nlm.nih.gov/geo/query/acc.cgi?acc=G. Source data are provided with this paper. Source data for Figs. 1–6 and Extended Data Fig. 1–8 have been provided as Source Data files. All other data supporting the findings of this study are available from the corresponding author on reasonable request.

## Source data

Source Data Fig. 1 Statistical source data.
Source Data Fig. 2 Statistical source data.
Source Data Fig. 3 Statistical source data.
Source Data Fig. 4 Statistical source data.
Source Data Fig. 5 Statistical source data.
Source Data Fig. 6 Statistical source data.
Source Data Extended Data Fig./Table 1 Statistical source data.
Source Data Extended Data Fig./Table 2 Statistical source data.
Source Data Extended Data Fig./Table 3 Statistical source data.
Source Data Extended Data Fig./Table 4 Statistical source data.
Source Data Extended Data Fig./Table 5 Statistical source data.
Source Data Extended Data Fig./Table 6 Statistical source data.
Source Data Extended Data Fig./Table 7 Statistical source data.
Source Data Extended Data Fig./Table 8 Statistical source data.

## Code availability

All code packages used for RNA-seq data analyses are described in the ‘RNA-seq analysis’ section in Methods.
