# Supplementary material for: CD103+ regulatory T cells underlie resistance to radio-immunotherapy and impair CD8+ T cell activation in glioblastoma
Source: Nat Cancer. 2023 Apr 20;4(5):665–81. doi: 10.1038/s43018-023-00547-6 (PMC10212765; doi:10.1038/s43018-023-00547-6)
Supplement: Supplementary file 1 — Reporting Summary [file 43018_2023_547_MOESM1_ESM.pdf]

nature portfolio

Corresponding author(s): Leila Akkari

Last updated by author(s): Mar 19, 2023

Reporting Summary

Nature Portfolio wishes to improve the reproducibility of the work that we publish. This form provides structure for consistency and transparency in reporting. For further information on Nature Portfolio policies, see our Editorial Policies and the Editorial Policy Checklist.

Statistics

For all statistical analyses, confirm that the following items are present in the figure legend, table legend, main text, or Methods section.

n/a	Confirmed	
☐	☒	The exact sample size (*n*) for each experimental group/condition, given as a discrete number and unit of measurement
☐	☒	A statement on whether measurements were taken from distinct samples or whether the same sample was measured repeatedly
☐	☒	The statistical test(s) used AND whether they are one- or two-sided<br>*Only common tests should be described solely by name; describe more complex techniques in the Methods section.*
☐	☒	A description of all covariates tested
☐	☒	A description of any assumptions or corrections, such as tests of normality and adjustment for multiple comparisons
☐	☒	A full description of the statistical parameters including central tendency (e.g. means) or other basic estimates (e.g. regression coefficient) AND variation (e.g. standard deviation) or associated estimates of uncertainty (e.g. confidence intervals)
☐	☒	For null hypothesis testing, the test statistic (e.g. *F*, *t*, *r*) with confidence intervals, effect sizes, degrees of freedom and *P* value noted<br>*Give P values as exact values whenever suitable.*
☒	☐	For Bayesian analysis, information on the choice of priors and Markov chain Monte Carlo settings
☐	☒	For hierarchical and complex designs, identification of the appropriate level for tests and full reporting of outcomes
☒	☐	Estimates of effect sizes (e.g. Cohen's *d*, Pearson's *r*), indicating how they were calculated

Our web collection on statistics for biologists contains articles on many of the points above.

Software and code

Policy information about availability of computer code

Data collection	FACS isolated cell samples were sorted directly into RLT buffer (Qiagen) and preparation of RNA library and transcriptome sequencing was conducted by Novogene Co., LTD (Beijing, China).
Data analysis	For analyses, a raw count matrix was produced and loaded within the R environment (version 4.1.1). DESeq2 (version 3.14) was used to assess the differential gene expression between grouped samples using an absolute log2 fold change of 1 and a false discovery rate of 0.05. BioPlanet56, Jensen Compartments57, WikiPathways58, MSigDB59, KEGG60 and GO61 databases were used as a primary source for gene set over-representation analyses. Over-representation was assessed with the enrichR package62 to check whether an input set of genes significantly overlaps with annotated gene sets using a false discovery rate of 0.05. Gene set enrichment analysis (GSEA) was assessed with the GAGE package63, which uses the average of the absolute values of the per gene test statistics to account for both up- and down-regulation of the curated pathways.

For manuscripts utilizing custom algorithms or software that are central to the research but not yet described in published literature, software must be made available to editors and reviewers. We strongly encourage code deposition in a community repository (e.g. GitHub). See the Nature Portfolio guidelines for submitting code & software for further information.

Data

Policy information about availability of data

All manuscripts must include a data availability statement. This statement should provide the following information, where applicable:

- Accession codes, unique identifiers, or web links for publicly available datasets
- A description of any restrictions on data availability
- For clinical datasets or third party data, please ensure that the statement adheres to our policy

Whole exome sequencing and RNA-sequencing data that support the findings of this study have been deposited in the Gene Expression Omnibus (GEO) under accession codes GSE203260. The data-set derived from this resource that supports the findings of this study is available in https://www.ncbi.nlm.nih.gov/geo/query/acc.cgi?acc=G.

Source data for Fig. 1-6 and Extended Data Fig. 1-8 have been provided as Source Data files. All other data supporting the findings of this study are available from the corresponding author on reasonable request.

Human research participants

Policy information about studies involving human research participants and Sex and Gender in Research.

Reporting on sex and gender	The findings in our study do not apply to only one sex/gender. Sex/gender was not considered in the study design.
Population characteristics	Glioblastoma patient samples (primary and recurrent standard of care therapy- surgical resection, 60Gy fractionated radiotherapy and temozolomide chemotherapy) were obtained with with patient consent and banked at the NKI biobank and obtained through the CMFPB541 authorization.
Recruitment	Patients were not part of a clinical trial but were treated at the Antoni van Leewenhoek hospital by Dr Dieta Brandsma and Dr Gerben Borst. Patient consented to have their samples banked at the NKI-Biobank. Patients did not receive compensation
Ethics oversight	Study registered at the Medical Ethics Committee at the Antoni van Leewenhoek and MC Slotervaart hospitals

Note that full information on the approval of the study protocol must also be provided in the manuscript.

Field-specific reporting

Please select the one below that is the best fit for your research. If you are not sure, read the appropriate sections before making your selection.

☒ Life sciences ☐ Behavioural & social sciences ☐ Ecological, evolutionary & environmental sciences

For a reference copy of the document with all sections, see nature.com/documents/nr-reporting-summary-flat.pdf

Life sciences study design

All studies must disclose on these points even when the disclosure is negative.

Sample size	For animal studies, sample size was determined with power calculation based on the mean and standard deviation from previous experimental results, and an alpha of 0.05 and power of 0.8 were taken as a guideline in these analysis. For ex vivo analysis, no statistical methods were used to pre-determine sample sizes but our sample sizes are similar to those reported in previous publications (e.g. Akkari et al., Science Translational Medicine, 2020 and Quail et al., Science, 2016).
Data exclusions	No data was excluded from the study.
Replication	For all experiments, biological replicates were used to guarantee reproducibility was ensured, with an n of at least 2-3
Randomization	Tumor volume of animals was determined by MRI and tumor size-matched mice were randomized over treatment groups (e.g. Control, RT, RT +IT).
Blinding	For animal experiments it was difficult to perform blinded treatments, because of the nature of the mouse treatments applied in experimental groups (e.g. radiation therapy, immunotherapy), However, the measure of tumor volumes was performed blindly as per which group of treatment the animals belong to. Data analysis on collected and digested tissue was done blindly. Ex vivo data analysis was done blindly. Human data analyses was performed blinded by a third party.

Reporting for specific materials, systems and methods

We require information from authors about some types of materials, experimental systems and methods used in many studies. Here, indicate whether each material, system or method listed is relevant to your study. If you are not sure if a list item applies to your research, read the appropriate section before selecting a response.

Materials & experimental systems

n/a	Involved in the study
☐	☒ Antibodies
☐	☒ Eukaryotic cell lines
☒	☐ Palaeontology and archaeology
☐	☒ Animals and other organisms
☒	☐ Clinical data
☒	☐ Dual use research of concern

Methods

n/a	Involved in the study
☒	☐ ChIP-seq
☐	☒ Flow cytometry
☐	☒ MRI-based neuroimaging

Antibodies

Antibodies used

All antibodies, dilutions and catalog numbers used in this study are listed in suppl. table 13 (referred to in the methods section), provided when submitting this paper.
Antibodies used in in vivo studies:
Antibodies against PD-1 (114111) and rIgG2a isotype control (RTK2758) were purchased from Biolegend. Antibody against CD25 (special order) was purchased from Evitrea. Antibodies against CD8 (BE0061), CTLA-4 (BE0164), mIgG2a isotype control (BP0085), rIgG2b isotype control (BP0090) and mIgG2b isotype control (BP0086) were purchased from Bioxcell.
Antibodies used for human flow cytometry:
Antibodies against CD11B (101251), CD11c (301635), CD123 (306034), CD127 (IL-7Ra) (351332), CD137 (309832), CD14 (325603), CD16 (302045), CD25 (302605), CD3 (300328), CD4 (317436), CD40 (334330), CD45 (304024), CD49d (304311), CD56 (318348), CD66B (305106), CD86 (374208), CD8a (300914), HLA-DR (307644), PD-L1 (329740), Tim3 (345018), and TNFRII (CD120b) (358412) were purchased from Biolegend. Antibodies against CD39 (564726), CD39 (564364) and Ki67 (563756) were purchased from BD Biosciences. Antibodies against CD19 (35-0198-42), FOXP3 (17-4776-41) and LAG3 (11-2239-42) were purchased from Invitrogen.
Antibodies used for mouse flow cytometry:
Antibodies against CD16/32 FC block (553142), B220 (CD45R) (563892), CD11b (563402), CD19 (612971), CD25 (564424), CD4 (612900), CD45 (564279), CD62L (612833), CD8 (748535), GrzB (563388), Ki67 (563756), MHCII (I-A/I-E) (565254) and PD-1 (562584) were purchased from BD Biosciences. Antibodies against CD103 (121432), CD103 (121433), CD11b (101243), CD11c (117329), CD140a (135914), CD19 (115546), CD3 (100326), CD4 (100550), CD40 (124618), CD44 (103047), CD45 (103128), CD49d (103618), CD64 (139306), CD69 (104536), CD80 (104738), CD86 (105036), GrzA (149704), GrzB (372208), ICOS (313518), IFNγ (505826), KLRG1 (106310), Ly6C (128036), Ly6G (127614), MHCI (H2-Kb) (116520), MHCI-SIINFEKL (141608), NK1.1 (108714), and PD-L1 (124308) were purchased from Biolegend. Antibody against OVA (AO1076a) was purchased at Abcepta. Antibody against Tim3 (DMABT-H26710) was purchased at Creative Diagnostics. Antibodies against CD25 (35-0251-8) and CD3 (46-0033-82) were purchased at eBioscience. Antibodies against CD24 (12702627), CD39 (67-0391-82), IFNγ (48-7311-82), and Lag3 (56-2239-42) were purchased at Fisher Scientific. Antibodies against CD8 (11-0081-82) and FOXP3 (35-5773-82) were purchased at Life Technologies. Antibodies against CD8 (46-0081-82) and iNOS (53-5920-82) were purchased at Thermofisher.
Antibodies used for immunohistochemistry:
Antibodies against B220 (557390) was purchased at BD Biosciences. Antibodies against CD3 (RM-9107-S1), CD4 (14-9766-82), CD8 (14-0808-82) and FOXP3 (14-5773) were purchased at Thermofisher. Antibody against Ki67 (K4003) was purchased at Agilent. Antibody against PD-1 (84651S) was purchased at Cell Signaling. Antibody against PNA (53-5920-82) was purchased at Vector laboratories.

Validation

The validation of each primary antibody for the species and application is available from manufacturers (statements on manufacturer's website). In addition, we validated our antibodies with fluorescence minus one (FMO) controls and titration experiments.

Eukaryotic cell lines

Policy information about cell lines and Sex and Gender in Research

Cell line source(s)

DF1 chicken fibroblasts were obtained from the American Type Culture Collection (ATCC). GL261 cells were provided as a kind gift from Prof. Johanna Joyce' lab.

Authentication

None of the cells were authenticated.

Mycoplasma contamination

All cell lines were routinely tested negative for mycoplasma contamination.

Commonly misidentified lines
(See ICLAC register)

No commonly misidentified cell lines were used in this study.

Animals and other research organisms

Policy information about studies involving animals; ARRIVE guidelines recommended for reporting animal research, and Sex and Gender in Research

Laboratory animals	Nestin-Tv-a;Ink4a/Arf-/- mice (BL/6 background) and Nestin-Tv-a mice (BL/6 background) were bred within the Netherlands Cancer Institute (NKI) animal facility. C57BL/6JRj mice were bred and delivered by Janvier labs. Male and female mice were used for experimental use at an age of 4-20 weeks. Housing conditions for the mice are IVC cages with add libitum food and water. The bedding material is corn cobs and new provided nesting material. There are 12 hour dark (7am-7pm) and 12 hour light cycles (7pm-7am). The temperature is 21 +/- 2 degrees and humidity is 55% +/-5%.
Wild animals	This study did not involve wild animals
Reporting on sex	The findings in our study do not apply to only one sex. Sex was not considered in the study design.
Field-collected samples	This study did not involve samples collected from the field
Ethics oversight	All mouse procedures were approved by the animal ethics committee of the Netherlands Cancer Institute and performed in accordance with institutional, national and European guidelines for animal care and use.

Note that full information on the approval of the study protocol must also be provided in the manuscript.

Flow Cytometry

Plots

Confirm that:

☒ The axis labels state the marker and fluorochrome used (e.g. CD4-FITC).

☒ The axis scales are clearly visible. Include numbers along axes only for bottom left plot of group (a 'group' is an analysis of identical markers).

☒ All plots are contour plots with outliers or pseudocolor plots.

☒ A numerical value for number of cells or percentage (with statistics) is provided.

Methodology

Sample preparation	Tissues were collected in ice-cold PBS and blood was collected in heparin-containing tubes. Tumors were macroscopically dissected and all non-tumor brain tissue was removed, unless otherwise stated in the figure legends. Blood samples were collected in Potassium/EDTA coated tubes and erylysis was performed for 10 minutes using lysis buffer (8,4 grams NH_4Cl + 1,2 gram $NaHCO_3$ + 0,2 ml 0,5M EDTA in 1 litre PBS). Cranial lymph nodes were digested by 3 mg ml−1 collagenase type A (Roche) and 25 µg ml−1 DNase (Sigma) in serum-free DMEM medium for 20min at 37°C. Single cell suspensions of brain tumors were obtained by enzymatic dissociation using a gentleMACS Octo Dissociator and the Tumor Dissociation kit (Miltenyi Biotec, Bergisch Gladbach, Germany). Tumor and lymph node cell suspensions were subsequently passed through a 40µm strainer (Corning, Sigma-Aldrich, St. Louis, MO, USA). Myelin depletion was then performed on tumor samples using Myelin Removal Beads II on MS columns (Miltenyi Biotec). Single cell suspensions were then subjected to Fc receptor blocking (rat anti-mouse CD16/32, BD Biosciences) for 15 min at 4°C and stained with conjugated antibodies for 30 min at 4° C in the dark in 2% FCS in PBS. Zombie NIR or Zombie Aqua (Biolegend) staining was performed to discriminate live and dead cells followed by fixation and permeabilization using the Cytofix/Cytoperm kit (BD Biosciences) in order to stain for intracellular proteins.
Instrument	Samples were acquired using a BD LSRFortessas TM (BD BioSciences) or a Cytek Aurora (Cytek) and cells were sorted using a FACSAria Fusion (BD BioSciences).
Software	Data analysis including quantification and data visualisation were performed using FlowJo Software version 10.7.1 (BD BioSciences) and Graphpad Prism 9.0.0 (Graphpad software).
Cell population abundance	Cells were gated according to well defined marker combination.
Gating strategy	All relevant gating strategies are described in the figure legends.

☒ Tick this box to confirm that a figure exemplifying the gating strategy is provided in the Supplementary Information.

Magnetic resonance imaging

Experimental design

Design type	Murine MRI-reported in methods and in previous publications (Akkari et al, Science Translational Medicine, 2020)
Design specifications	N/A

Behavioral performance measures	N/A

Acquisition

Imaging type(s)	T2 weight
Field strength	7T
Sequence & imaging parameters	N/A
Area of acquisition	Tumor region based on T2 weight contrast.

Diffusion MRI ☐ Used ☒ Not used

Preprocessing

Preprocessing software	MIPAV
Normalization	N/A
Normalization template	N/A
Noise and artifact removal	N/A
Volume censoring	N/A

Statistical modeling & inference

Model type and settings	N/A
Effect(s) tested	N/A

Specify type of analysis: ☐ Whole brain ☒ ROI-based ☐ Both

Anatomical location(s): Brain

Statistic type for inference (See Eklund et al. 2016)	Voxel wise
Correction	N/A

Models & analysis

n/a	Involved in the study
☒	☐ Functional and/or effective connectivity
☒	☐ Graph analysis
☒	☐ Multivariate modeling or predictive analysis
